# Molecular determinant of substrate binding and specificity of cytochrome P450 2J2

**DOI:** 10.1038/s41598-020-79284-0

**Published:** 2020-12-17

**Authors:** Liang Xu, Liao Y. Chen

**Affiliations:** Department of Physics and Astronomy, University of Texas at San Antonio, One UTSA Circle, San Antonio, TX 78249 USA

**Keywords:** Biophysics, Computational biology and bioinformatics, Structural biology

## Abstract

Cytochrome P450 2J2 (CYP2J2) is responsible for the epoxidation of endogenous arachidonic acid, and is involved in the metabolism of exogenous drugs. To date, no crystal structure of CYP2J2 is available, and the proposed structural basis for the substrate recognition and specificity in CYP2J2 varies with the structural models developed using different computational protocols. In this study, we developed a new structural model of CYP2J2, and explored its sensitivity to substrate binding by molecular dynamics simulations of the interactions with chemically similar fluorescent probes. Our results showed that the induced-fit binding of these probes led to the preferred active poses ready for the catalysis by CYP2J2. Divergent conformational dynamics of CYP2J2 due to the binding of each probe were observed. However, a stable hydrophobic clamp composed of residues I127, F310, A311, V380, and I487 was identified to restrict any substrate access to the active site of CYP2J2. Molecular docking of a series of compounds including amiodarone, astemizole, danazol, ebastine, ketoconazole, terfenadine, terfenadone, and arachidonic acid to CYP2J2 confirmed the role of those residues in determining substrate binding and specificity of CYP2J2. In addition to the flexibility of CYP2J2, the present work also identified other factors such as electrostatic potential in the vicinity of the active site, and substrate strain energy and property that have implications for the interpretation of CYP2J2 metabolism.

## Introduction

**Figure 1:**
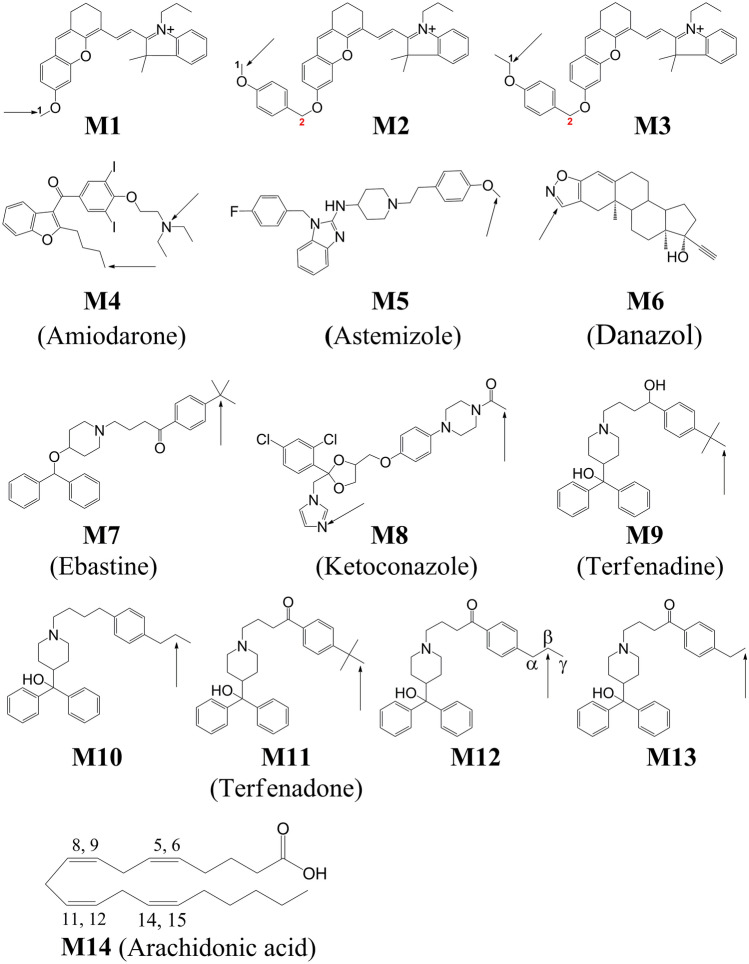
Molecular structures of **M1**–**M14** used in this work. The C^1^ in **M1**–**M3** indicated the actual metabolic site catalyzed by CYP2J2 whereas the C^2^ in **M2** and **M3** was the metabolic site predicted by current molecular docking studies. The potential metabolic sites of **M4**–**M14** was indicated by an arrow (**M4**–**M13**) or labels (**M14**). The metabolic site of **M1**–**M3** was adopted from reference^25^; The metabolic site of M4 was adopted from reference^14^; The metabolic site of M5 was adopted from reference^10^; The metabolic site of M6 was adopted from reference^13^; The metabolic site of M7 was adopted from reference^11^; The metabolic site of M8 was adopted from CYP3A4 in complex with two M8 (PDB ID: 2J0C, see reference^23^) and reference^26^; The metabolic site of M9–M13 was adopted from reference^12^; and The metabolic site of M14 was adopted from reference^6^.

Cytochrome P450 (CYP) enzymes constitute a diverse group of heme-containing proteins, which play pivotal roles in the metabolism of xenobiotics including a wide variety of drugs, and the conversion of polyunsaturated fatty acids (PUFAs) to biologically active molecules^1,2^. The crystal structures of a large number of CYPs reveal that CYPs share a similar overall protein topology but exhibit varying substrate selectivity^2,3^. The molecular basis for this unusual specificity of CYPs could be attributed to the plasticity of the substrate-binding pocket near the heme motif that enables a specific CYP to accommodate one or two substrates in its active site^4^. Among 57 CYPs identified in human^5^, the 2J2 isoform (CYP2J2) mediates lipid metabolism of PUFAs via epoxidation reactions, yielding the epoxyeicosatrienoic acids (EETs). For instance, in human heart, the CYP2J2 enzyme metabolizes arachidonic acid (ARA, **M14**, Fig. 1) predominantly via olefin epoxidation to all four regioisomeric EETs, with epoxidation occurring preferentially at the 14,15-olefin (37% of total EET products), followed by 11,12-EET (18%), 8,9-EET (24%), and 5,6-EET (21%)^6^. The metabolism of ARA to EETs seems to relate to the cardioprotective role of CYP2J2^7^, but could be influenced by the metabolism of drugs that competitively or noncompetitively inhibit the EET synthesis and consequently lead to cardiotoxicity^8^. In addition to cardiovascular disease, CYP2J2 is also implicated in cerebrovascular diseases, diabetes, and cancer^9^.

Besides endogenous substrates, CYP2J2 has been reported to metabolize some antihistamine drugs such as astemizole (**M5**, Fig. 1)^10^, ebastine (**M7**, Fig. 1)^11^, and terfenadine (**M9**, Fig. 1)^12^. Several new substrates including tamoxifen, albendazole, danazol (**M6**, Fig. 1), amiodarone (**M4**, Fig. 1), and ketoconazole (**M8**, Fig. 1) were also identified^13–16^. Interestingly, danazol was shown to strongly inhibit the CYP2J2-mediated metabolism of albendazole, astemizole, ebastine, and terfenadine in a substrate-independent manner^17^. Further studies suggested that the inhibitory effects of danazol depended on the human microsome used^18^, and terfenadone (**M11**, Fig. 1) was proposed as a general CYP2J2 inhibitor in both human liver and intestinal microsomes^18^. Despite its critical role in drug metabolism, the lack of crystal structure of CYP2J2 hampers a better understanding of its adaptation to chemically diverse compounds. The emerging computational modeling and simulations provided invaluable insights into the structure of CYP2J2 and corresponding substrate interactions. Several CYP2J2 homology models have been constructed based on single^19–21^ or multiple templates^12,13,22^. These templates are primarily CYP gene in group 2 (CYP2) showing varying sequence identity (26–46%) with CYP2J2. A brief summary of these published models was provided by Proietti et al.^21^. When used to interpret the protein-substrate interactions, it seems challenging to achieve consistent results with structural models generated using different protocols. For instance, based on the CYP2J2 homology model developed using multiple CYP2 template proteins (2A6, 2B4, 2C5, 2C8, and 2D6), one terfenadone derivative (**M12**, Fig. 1) was predicted to interact with Arg117 through its keto (C = O) group^12^, whereas such an interaction was not found in another study where a CYP2J2 model was constructed based on the crystal structure of CYP2C9^19^. The difficulty in the prediction of the preference of metabolic site of ARA was demonstrated in two studies of interactions between CYP2J2 and ARA. Based on the CYP2J2 model generated using the template of CYP2R1, ARA was predicted to form hydrogen bonds with Gly486 and Leu378 in its favorable binding mode^20^. However, these hydrogen bond interactions were not observed in another study using the CYP2J2 model built based on the crystal structure of rabbit CYP2B4, and the preferred binding pose that yielded the metabolic product of 13,14-EET was not predicted^21^. In addition, the binding modes of the above-mentioned antihistamine drugs in the active site of those developed CYP2J2 models were not carefully examined. The multiple ligand binding modes and the conformational dynamics induced by ligand binding were observed in CYP3A4, indicating the structural flexibility in CYP enzymes^23^. On the other hand, CYP3A4 could regioselectively metabolize chemically diverse drugs, suggesting specific protein-substrate interactions in the active site of CYP enzymes^24^. The substrate promiscuity in CYP enzymes challenges the attempt to identify the molecular determinant of the substrate specificity in the active site of CYP2J2.

**Figure 2:**
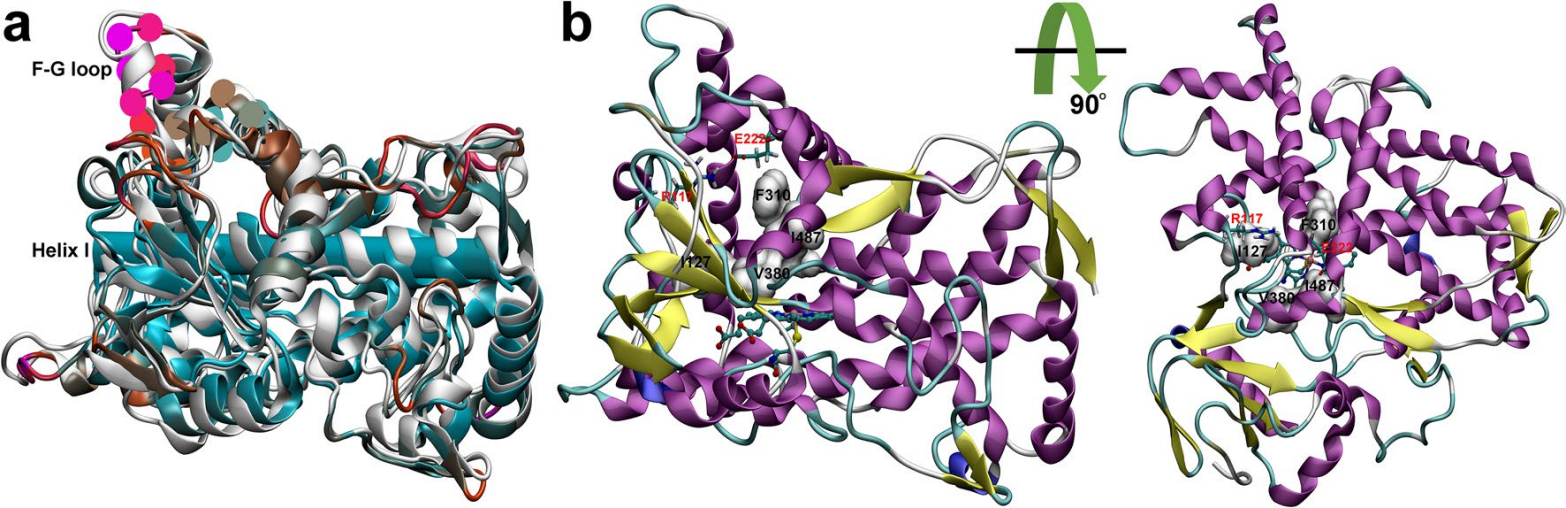
(**a**) Structural superimposition of initial (white color) and the representative conformations of CYP2J2. The helix I composed of residues 297–330 was also shown in Cartoon representation. The mobile F-G loop formed by residues 224–240 was highlighted by spheres. The flexibility increased from the cyan region (inflexible) to the orange and magenta regions (most flexible). (**b**) Representative conformation of CYP2J2 in side and top views, obtained from the 500-ns MD simulations of ligand-free CYP2J2. The key residues Ile127, Phe310, Ala311, Val380, and Ile487 were shown in white spheres (hydrophobic residues). The heme moiety was shown in CPK representation. Arg117 and Glu222, which formed a stable salt-bridge during simulations, were also highlighted in Licorice representation and labeled in red color. This figure was rendered using VMD^27^ (http://www.ks.uiuc.edu/Research/vmd/).

In this work, we revisited the structure of CYP2J2 by homology modeling based on multiple templates, and fully relaxed the model structure by performing 500-ns molecular dynamics (MD) simulations in aqueous solution (Fig. 2). Three newly reported fluorescent probes (**M1**–**M3**, Fig. 1)^25^ were used to investigate the protein–ligand interactions and conformational dynamics of CYP2J2 upon ligand binding. The CYP2J2-mediated *O*-demethylation (C^1^ in **M1**–**M3**, Fig. 1) and subsequent 1,6-elimination of *p*-hydroxybenzyl resulted in the probe that produced a strong near-infrared fluorescence signal^25^. These compounds are structurally similar and suitable to study the difference in the ligand binding modes caused by the subtle variations in the ligand structures. The present simulation results showed that these three probes displayed distinct interactions with CYP2J2 in the binding pocket. The probe **M2** showing the highest fluorescence response toward CYP2J2 was predicted to have the highest binding affinity for CYP2J2. The molecular determinant for the access of any substrate to the active center of CYP2J2 was identified based on our computational simulation and docking results. The structural information obtained from the present study sheds light on the substrate specificity mediated by interactions with CYP2J2, and may have implications for drugs metabolized by CYP2J2.

## Results

### Induced-fit binding of M1–M3 to CYP2J2 resulted in active conformers

**Figure 3:**
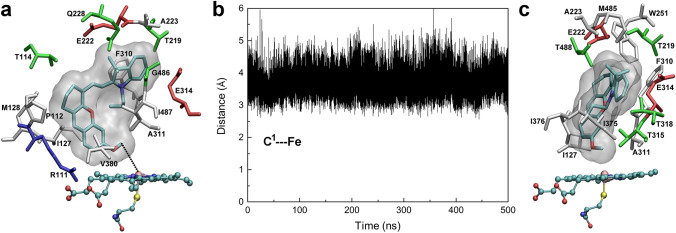
MD simulations of CYP2J2 in complex with **M1**. (**a**) The initial binding pose of **M1** in the active site of CYP2J2; (**b**) The distance between the proposed metabolic site (C^1^ atom) and the heme iron during the simulations; and (**c**) The representative binding conformation of **M1** in the active site of CYP2J2. Interacting residues with a contact frequency > 50% calculated from the last 100-ns trajectory were shown and colored based on their types: blue for basic, red for acidic, green for polar, and white for nonpolar residues. A contact occurs if any atom of the ligand is within 3 Å of any atom of protein residues. This figure was rendered using VMD^27^ (http://www.ks.uiuc.edu/Research/vmd/).

**Figure 4:**
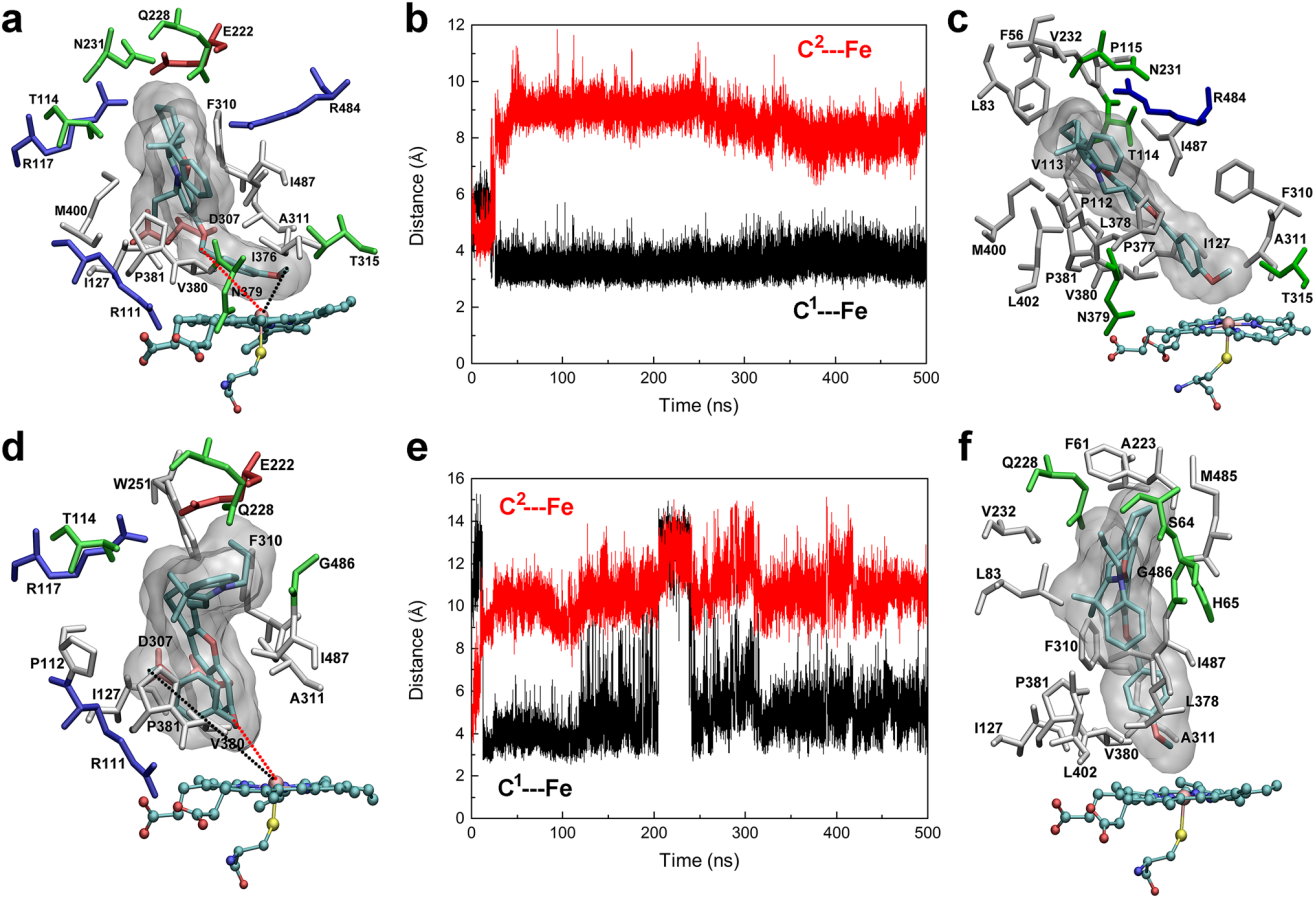
MD simulations of CYP2J2 in complex with **M2** in the extended (**a**–**c**) or folded conformation (**d**–**f**). (**a**, **d**) The initial binding pose of **M2** in the active site of CYP2J2; (**b**, **e**) The distance between the proposed metabolic site (C^1^ atom) and the heme iron during the simulations. For comparison, the distance between another C^2^ atom and the heme iron was also monitored; and (**c**, **f**) The representative binding conformation of **M2** in the active site of CYP2J2. This figure was rendered using VMD^27^ (http://www.ks.uiuc.edu/Research/vmd/).

**Figure 5:**
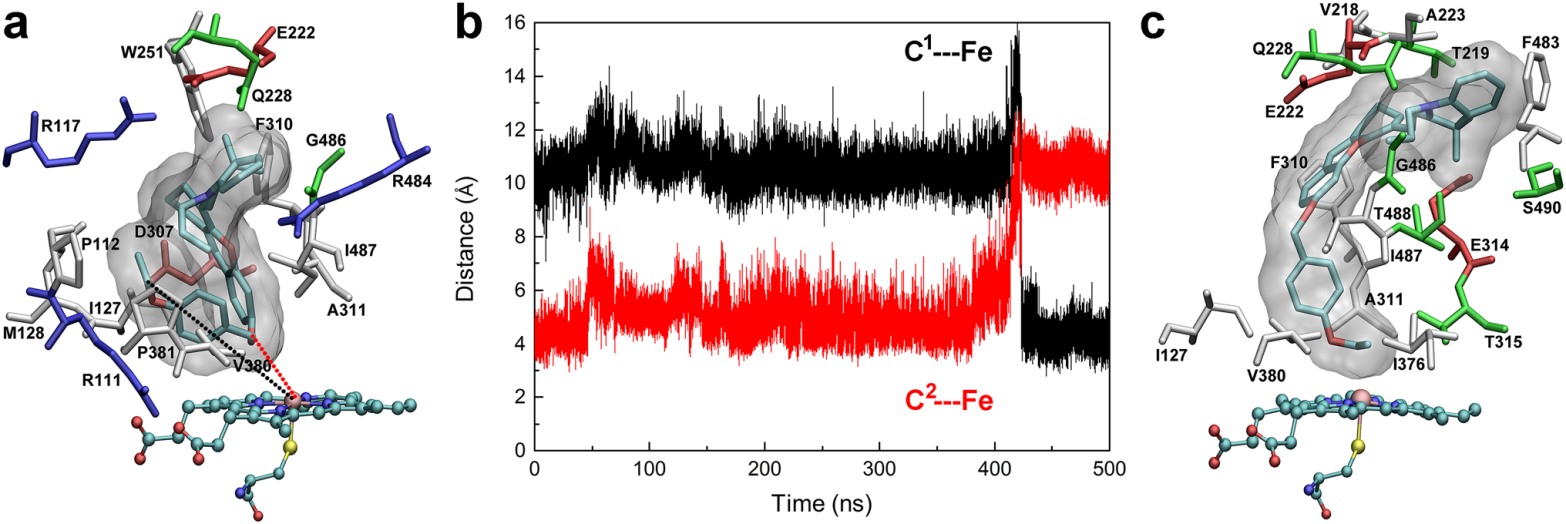
MD simulations of CYP2J2 in complex with **M3**. (**a**) The initial binding pose of **M3** in the active site of CYP2J2; (**b**) The distance between the proposed metabolic site (C^1^ atom) and the heme iron during the simulations. For comparison, the distance between another C^2^ atom and the heme iron was also monitored; and (**c**) The representative binding conformation of **M3** in the active site of CYP2J2. This figure was rendered using VMD^27^ (http://www.ks.uiuc.edu/Research/vmd/).

The stability and structural fluctuations of the CYP2J2 structure were demonstrated by the root-mean-square deviation (RMSD) and root-mean-square fluctuation (RMSF) values over the 500-ns simulations (Fig. S1 under Supplementary Information). After 300-ns dynamics, the structure seemed well equilibrated with the RMSDs below 2.0 Å and RMSFs below 2.5 Å. For CYP2J2 in complex with **M1**/**M2**/**M3**, the RMSDs were less than 2.5 Å. The large fluctuations in the RMSFs appeared in **M2**-bound CYP2J2. The binding of **M2** in the extended (M2a) and folded conformer (M2b) resulted in more flexible F-G loop composed of residues 224–240 and the N-terminus, respectively. The conserved helix I region composed of residues 290–330 remained relatively stable (Fig. 2 and Fig. S1). The volume of the binding pocket of the representative conformation of CYP2J2 was calculated using the MDpocket software^28^, and a value of 1499 Å^3^ was obtained, which seemed large enough to accommodate **M1** and **M2** in their extended conformer. As shown in Figs. 3a and 4a, the initial binding pose of **M1** and **M2** displayed the optimal distance between the catalytic site (C^1^ in **M1**–**M3**, Fig. 1) and the heme iron. However, for **M3**, such an extended conformer was not found in the current docking study, and a folded conformer with the minimum distance between another carbon atom (C^2^ in **M3**, Fig. 1) and the heme iron was selected as the initial binding conformation (Fig. 5a). For comparison, a similar folded binding conformer of **M2** was also simulated (Fig. 4d). The overlapping of the initially folded binding conformers of **M2** and **M3** was shown in Fig. S2, indicating similar binding poses.

The distance between the catalytic site of **M1** and the heme iron fluctuated marginally from the initial 3.9 Å to a value of 3.7 ± 0.2 Å block-averaged over the last 100-ns simulations (Fig. 3b), implying a relatively stable binding of **M1** to CYP2J2. Because of the induced-fit binding^29^, the interactions of **M1** with CYP2J2 altered the orientation of **M1** in the active site, which was demonstrated by the changes in the interacting residues. Residues including Arg111, Pro112, Thr114, Met128, Gln228, Val380, Gly486, and Ile487 that were observed in the initial binding conformation were not identified to interact with **M1** during the simulations. Several new residues such as Trp251, Thr315, Thr318, Ile375, Ile376, Met485, and Thr488 were found to be involved in the interactions with **M1**. And residues including Ile127, Thr219, Glu222, Ala223, Phe310, Ala311, and Glu314 persistently interacted with **M1**.

Two binding poses of **M2** in the active site of CYP2J2 were investigated, with **M2** in either extended (Fig. 4a–c) or folded conformer (Fig. 4d–f). Except for the large fluctuations during the first 50-ns simulations, the compound **M2** with an initially extended structure seemed adapted to the binding environment of CYP2J2, as the distance between the catalytic site and the heme iron was well maintained, and an average value of 3.7 ± 0.1 Å was obtained based on the last 100-ns simulations. Similar to **M1**, the induced-fit binding of **M2** also mediated its interactions with CYP2J2. More preserved residues were identified including Thr114, Ile127, Asn231, Phe310, Ala311, Thr315, Asn379, Val380, Pro381, Met400, Arg484, and Ile487.

Interestingly, when **M2** bound to CYP2J2 in a folded state (Fig. 4d–f), it quickly changed its conformation from the initially deformed state to the extended state, probably because the deformed binding pose could cause large strain energy that may not be compensated by the binding interactions with the protein^30^. As a consequence, **M2** adopted a different binding pose that was induced by the interactions with CYP2J2. And the distance between the catalytic site and the heme iron decreased from the initial 10 Å to 5.0 ± 0.2 Å (block-averaged over the last 100-ns simulations). Although **M2** underwent significant conformational changes, there were still eight interacting residues preserved, including Ile127, Gln228, Phe310, Ala311, Val380, Pro381, Gly486, and Ile487.

In the case of **M3** where it adopted a deformed conformation similar to that of **M2** initially (Fig. S2), it took **M3** a longer time (~ 200 ns) to adopt the extended conformation in the binding pocket of CYP2J2; then approach to the active site after 423 ns; and remain in the bound state (Fig. 5 and Fig. S3) till the end of the simulations. The calculated distance between the catalytic site and the heme iron was 4.3 ± 0.1 Å (block-averaged over the last 50-ns simulations). The preserved interacting residues included Ile127, Glu222, Gln228, Phe310, Ala311, Val380, Gly486, and Ile487.

### Structural basis for the high binding affinity of M2 to CYP2J2

The *O*-alkyl group in the structure of **M1**–**M3** was supposed to be the metabolic site. And **M1** showed rather weak fluorescence response, indicating that **M1** could hardly be catalyzed by CYP2J2^25^. With the introduction of the linker (*p*-hydroxybenzyl) to **M1**, **M2** displayed the highest fluorescence response toward CYP2J2. Replacing the *O*-methyl group in **M2** with *O*-ethyl group led to **M3**, which significantly reduced the CYP2J2-mediated *O*-demethylation^25^. From our simulations, it was found that these three probes were capable of binding to the catalytic site of CYP2J2, especially for **M2** and **M3** even when their initial binding poses deviated from the final active conformers, indicating a high binding preference of **M2** and **M3** to CYP2J2. The distance from the heme iron to the metabolic site of the substrate was assumed to be related to the rate of metabolism of CYP2J2^12,31^. Based on this assumption, **M1** (3.7 ± 0.2 Å) should exhibit a higher reactivity than **M3** (4.3 ± 0.1 Å), which seemed to contradict the experimental results.

The distance between the catalytic site of substrates to the heme iron varies in different CYP enzymes. For instance, a distance of 2.1 Å was observed in the crystal structure of CYP3A4 in complex with ketoconazole (PDB ID: 2J0C)^23^; a larger distance of 4.4 Å was observed in another crystal structure of CYP3A4 in complex with midazolam (PDB ID: 5TE8)^24^; and an even larger distance of 6.6 Å was found in the crystal structure of CYP2C9 (PDB ID: 5W0C) in complex with one inhibitor and water molecules in the active site^32^. In the system where the distance from **M2** to heme iron was 5.0 ± 0.2 Å, water molecules were also found to transiently bind to heme, and may prevent **M2** from moving closer to the active site of CYP2J2 (Fig. S4). Taken together, it appeared that the distances between **M1**–**M3** and heme iron were in a reasonable range, and these three probes could be catalyzed by CYP2J2. However, the difference in their catalytic efficiency (**M2** > **M3** > **M1**) implied that the distance may not be the determining factor for substrate metabolism.

**Figure 6:**
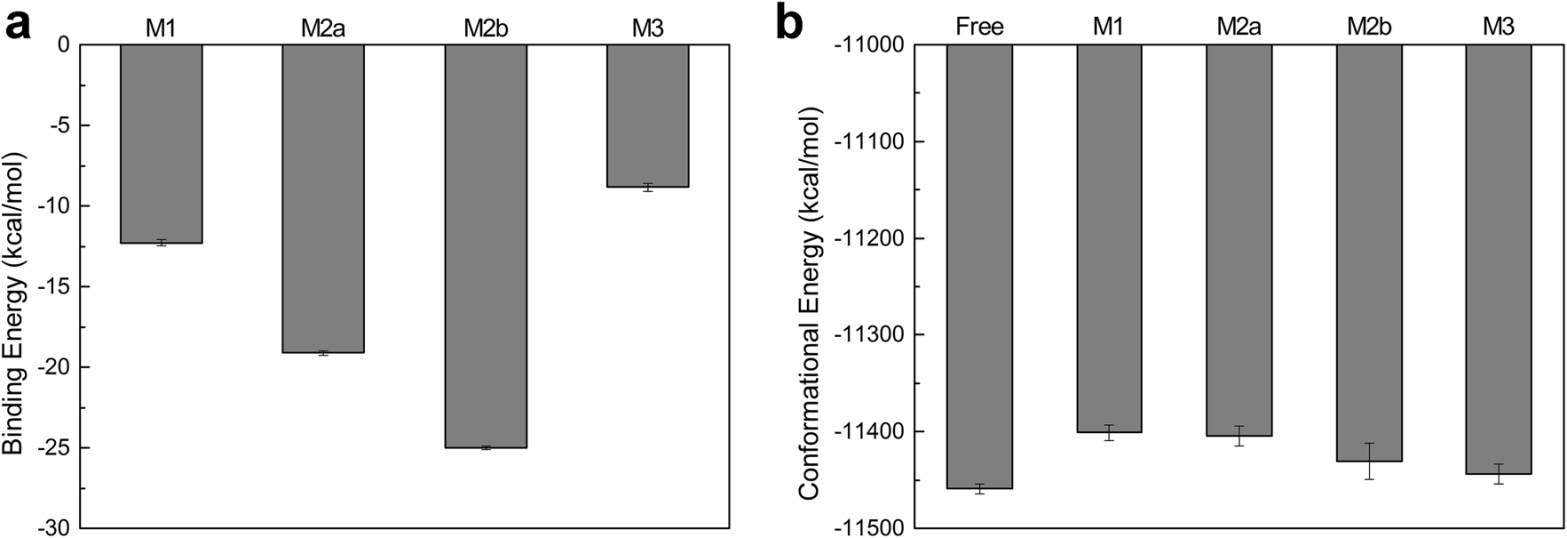
The binding energy of **M1**–**M3** to CYP2J2 (**a**) and conformational energy of CYP2J2 in complex with **M1**–**M3** (**b**). For comparison, conformational energy for the free CYP2J2 was also calculated. M2a and M2b denote initially extended and folded conformation of **M2** in the binding pocket of CYP2J2, respectively.

The estimated binding affinity of **M1**–**M3** to CYP2J2 was shown in Fig. 6. **M2** displayed the highest binding affinity to CYP2J2, regardless of their initially different binding poses. The finding that **M2** tended to adopt an extended conformation further suggested that little strain energy was induced when binding to CYP2J2. To test this, 100-ns MD simulations of **M1**/**M2**/**M3** in aqueous solution were performed, and it was found that these three molecules exclusively adopted an extended conformation in solution (Fig. S5). Compared to the unbound state in solvent, **M3** became deformed to some extent and the strain energy costs of restraining **M3** to the bound state was not taken into account in the binding energy calculations, which might result in substantial uncertainty in the calculated binding affinity of **M3** to CYP2J2.

**Figure 7:**
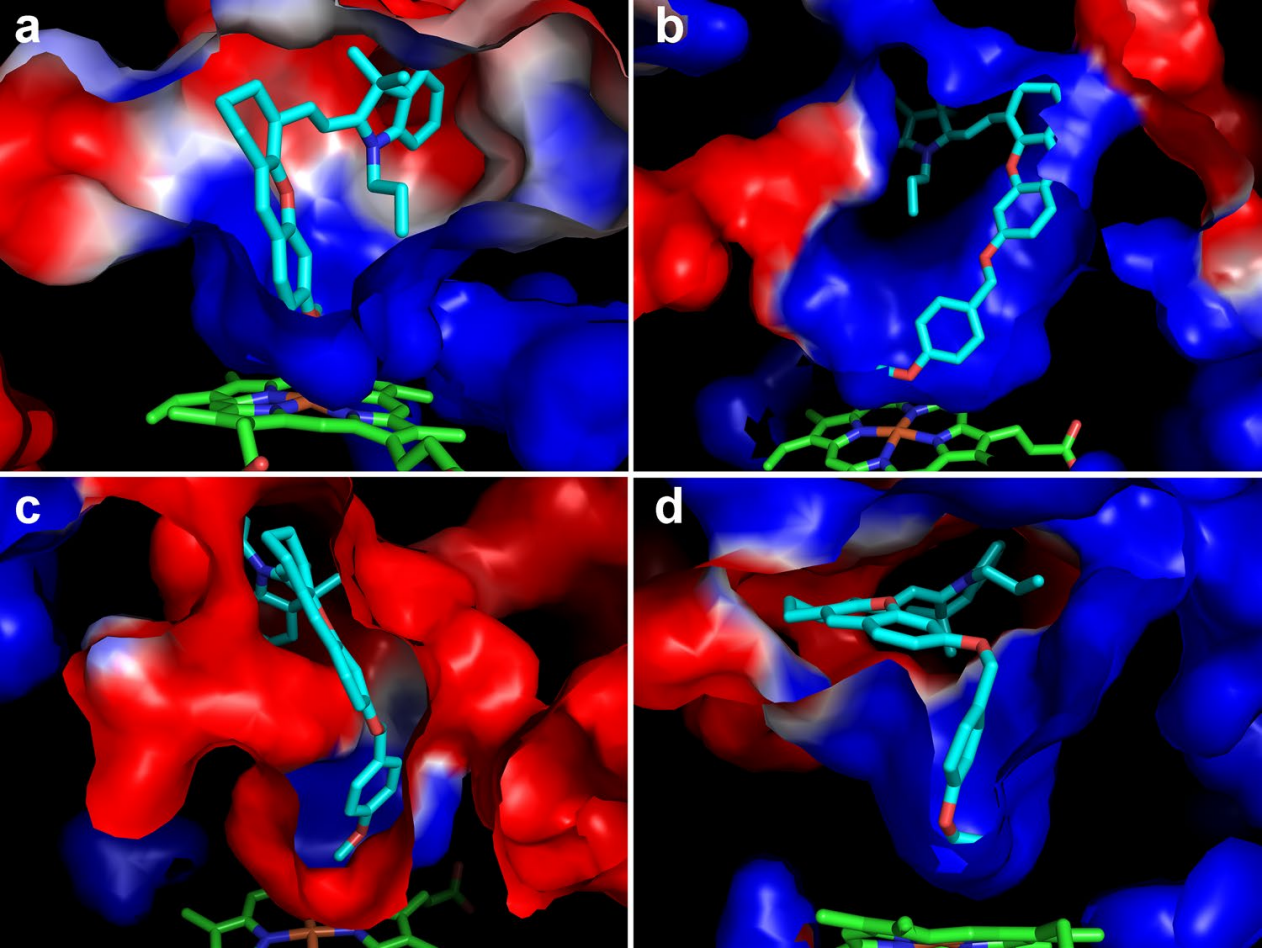
Electrostatic potential surface calculated for the representative conformation of CYP2J2 in complex with **M1** (**a**), **M2** in the initially extended (**b**) and folded (**c**) conformation, and **M3** (**d**). Blue color indicates positive electrostatic potentials, and red color indicates negative electrostatic potentials. This figure was rendered using PyMOL^33^.

The difference in the interacting residues in the vicinity of the active site could contribute to the differing binding energy of **M2** in different binding poses. Note that **M1**–**M3** were positively charged, to further examine the effects of these interacting residues to the ligand binding, the electrostatic potential surface was calculated for the structure of ligand-bound CYP2J2 using the APBS tool^34^ (Fig. 7). The presence of a significant area of negative electrostatic potential around **M2** provided another support for its highest binding affinity and catalytic efficiency (Fig. 7c). The presence of negative electrostatic potential was also observed in the vicinity of **M3**, especially around the positively charge moiety of **M3** (Fig. 7d). In contrast, for **M2** with a relatively lower binding affinity to CYP2J2, a large area of positive electrostatic potential in the vicinity of it was found (Fig. 7b), and the presence of both negative and positive potentials around **M1** was observed (Fig. 7a). The favorably negative potential was likely to play a critical role in stabilizing **M2** in the active site of CYP2J2. For comparison, we also calculated the electrostatic potential for the initial binding conformations (Fig. S6), none of them showed favorable potential for the binding of **M1**–**M3**, which may explain why **M1**–**M3** would undergo substantial changes in the binding poses during simulations. Because of the conformational dynamics of CYP2J2, we randomly selected another conformation for each system and calculated the electrostatic potential, and similar results were obtained (Fig. S7), confirming the presence of favorable electrostatic environment for the binding and catalysis of **M2** and **M3**. The present results were consistent with previous studies suggesting that in addition to the hydrophobic interactions, the electrostatic properties of the enzymes’ surroundings also influenced the binding of both charged and nonpolar ligands^35,36^.

### Interactions with M1–M3 induced divergent dynamics of CYP2J2

The effects of induced-fit binding of **M1**–**M3** on the conformational dynamics of CYP2J2 were characterized in terms of the conformational energy and the cross-correlated network maps of Cα atomic fluctuations using the R package Bio3D^37,38^. The calculated conformational energy suggested that the binding of **M1**–**M3** destabilized the conformations of CYP2J2 to different extents, compared to the free state of CYP2J2 (Fig. 6b). Of interest, less destabilization effects were observed in the two systems where **M2** and **M3** initially were deformed, implying the capability of CYP2J2 in the mediation of the interactions with the ligands. If **M2** bound to CYP2J2 with the extended conformation, it would become difficult for CYP2J2 to reshape the binding pocket and direct the binding of **M2** toward the conformations with less energy cost.

**Figure 8:**
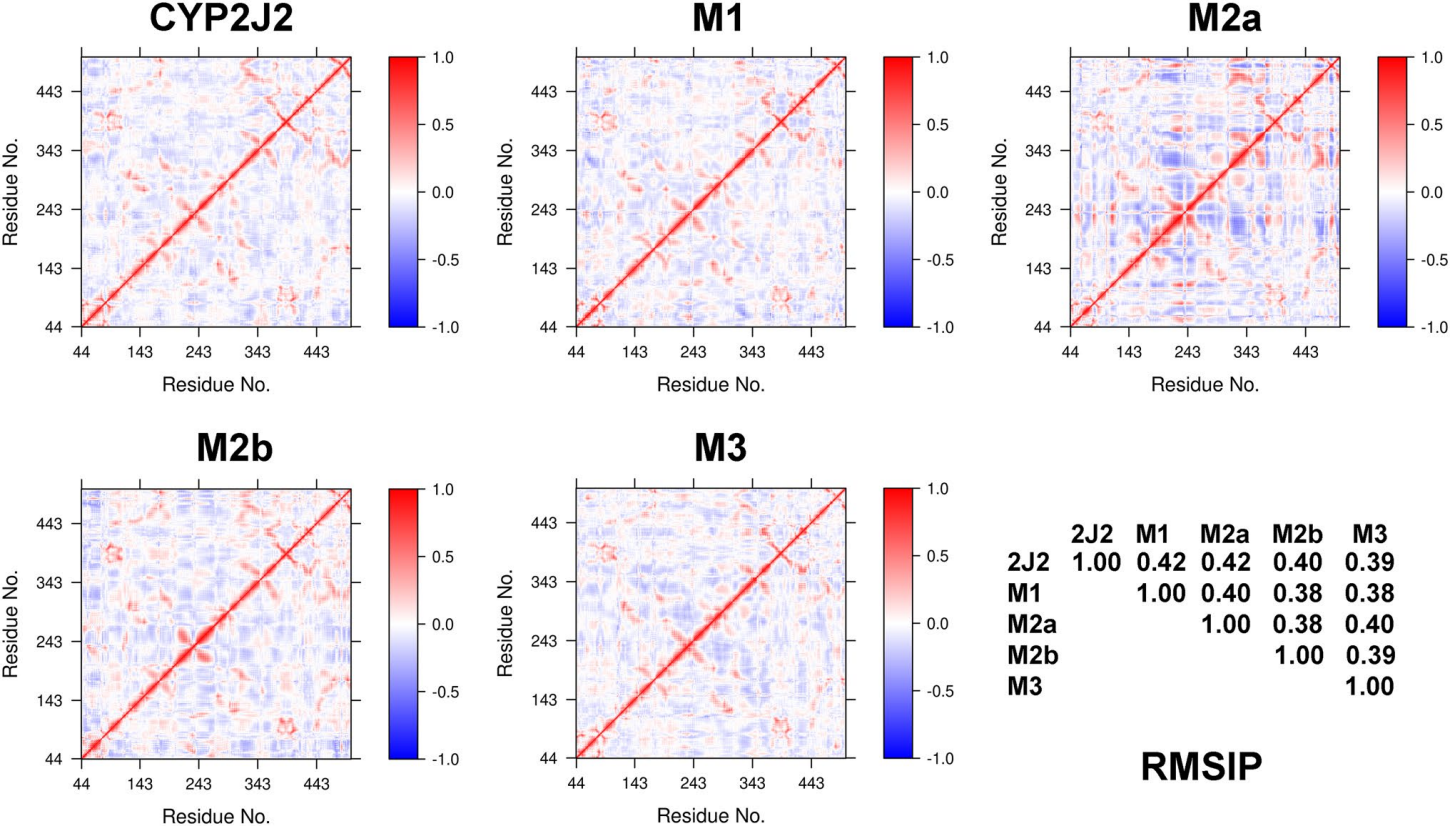
The dynamical cross-correlation maps (DCCMs) for the free and **M1**/**M2**/**M3**-bound CYP2J2. The root mean square inner product (RMSIP) between any two different systems was also calculated. M2a and M2b denote initially extended and folded conformation of **M2**, respectively. This figure was rendered using Bio3D^37^.

The dynamical cross-correlation maps (DCCMs) for the free and ligand-bound CYP2J2 were calculated from the MD simulations (Fig. 8). The cross-correlation coefficients vary from a value of 1 for completely correlated motions to − 1 for completely anticorrelated motions^39^. More anticorrelated motions were identified for the binding of **M2** with the initially extended conformation, consistent with its destabilization effect on the dynamics of CYP2J2. To quantitatively measure the amount of overlap between two conformations, the root mean square inner product (RMSIP) was calculated on the first 20 eigenvectors obtained from a principal component analysis of the Cα covariance matrix of the atomic positional fluctuations^40^. The value of RMSIP ranges from 0 for mutually orthogonal (no correlation) subspaces to 1 for identical subspaces (full correlation). A value of 0.5 is considered fairly correlated subspaces^41^. The calculated RMSIP values between any two different systems varied between 0.38 and 0.42, indicating significant dissimilarity in the conformational spaces sampled by CYP2J2 bound to different ligands.

One of interesting structural aspects of CYP450 is the substrate access channels that may modulate CYP450 function^42^. To demonstrate the impacts of the distinct conformational dynamics of CYP2J2 in complex with **M1**–**M3**, the potential substrate access channels were analyzed using CAVER^43^. The heme iron was chosen as the starting point to detect the potential access channels. For comparison, the substrate access channels in the human CYP2B6 in complex with two amlodipine molecules (PDB ID: 3UA5)^44^ were also calculated. In this structure, in addition to one substrate in the active site, another substrate was trapped in access pathway, and thus the positions of the two substrates clearly implied the substrate access channel (Fig. S8). Interestingly, a similar substrate access channel was found in CYP2J2 regardless of the presence or absence of the ligand, suggesting that **M1**–**M3** could use the same channel to reach the active site. In addition to the access channels, other channels that may serve as substrate (or metabolic product) exit channels were detected when there was no or little overlap with the access channel. It was found that a distinct exit channel was identified in the ligand-free CYP2J2 when compared with the exit channel in CYP2B6. And this channel seemed well maintained only in the conformation of CYP2J2 in complex with M2a. For the conformations of M2b- and **M3**-bound CYP2J2, all other detected channels overlapped with the access channel to different extents and therefore the exit channels seemed to be largely buried in the protein interior. The above results suggested that the conformational dynamics induced by substrates significantly influenced the channel networks, especially the potential substrate exit channels.

### Molecular determinant for the access of substrate to the active center of CYP2J2

Although **M1**–**M3** adopted distinct binding poses in the substrate pocket of CYP2J2, five interacting residues (Ile127, Phe310, Ala311, Val380, and Ile487) were identified to control the substrate access to the heme motif. The distances between these residues were calculated and shown in Fig. S9, and no significant deviations from the free CYP2J2 were observed, suggesting that these residues appeared to form a bottleneck that allowed the access of only one substrate to the heme motif. The binding of **M1**, however, resulted in notable increase of the distances associated with Ile487, but no interactions with Ile487 were found for **M1** (Fig. 3b, the actual contact frequency is 0.02%), suggesting that the binding of **M1** might distort this bottleneck. It remains to be elucidated if the catalytic rate of CYP2J2 would be related to the stability of these residues, but the maintenance of their positions in **M2** that exhibited the highest catalytic rate for CYP2J2 might indicate their important role in the metabolism of substrates.

**Figure 9:**
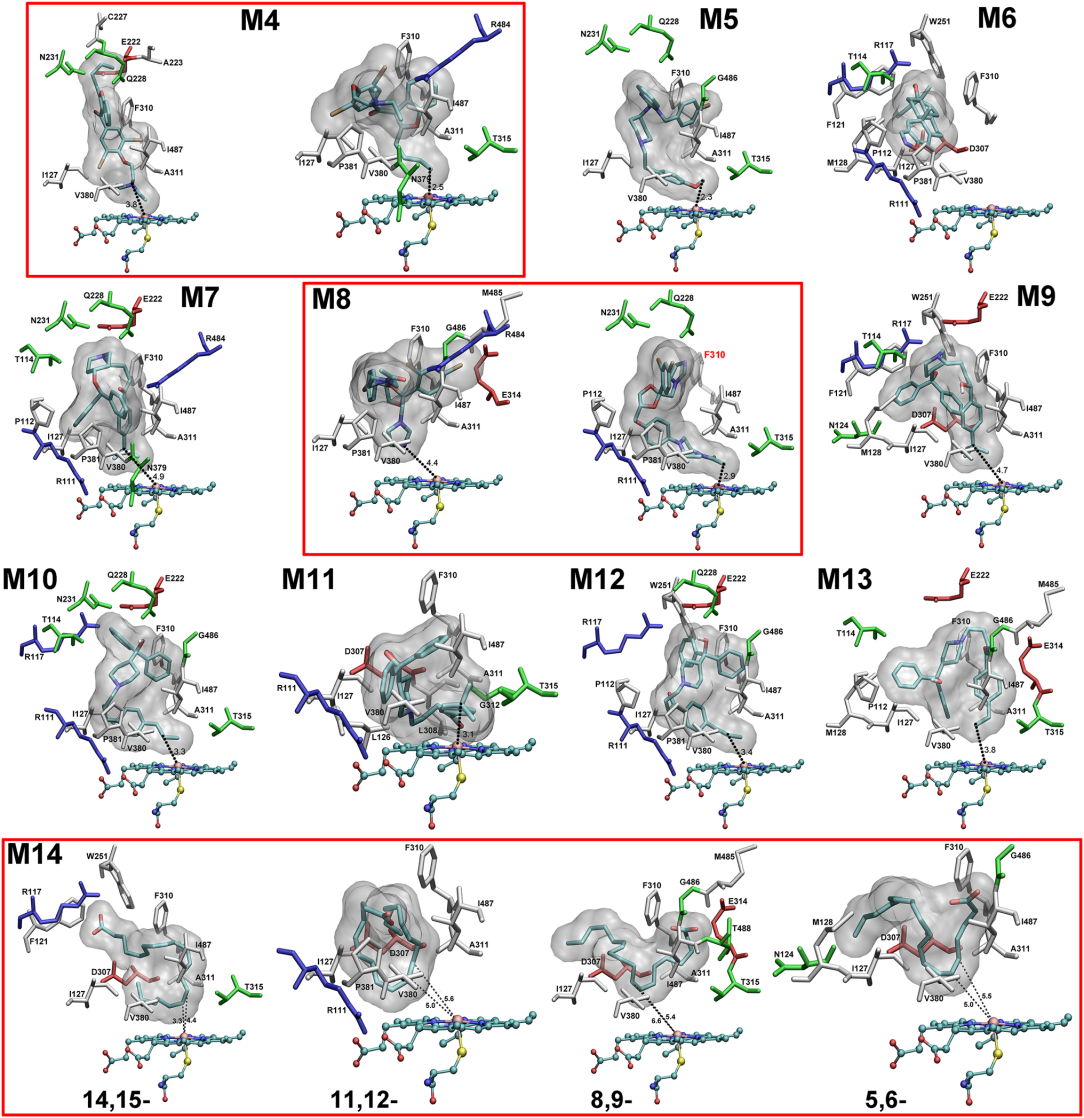
Summary of the preferred binding pose for **M4**–**M14** in the active site of CYP2J2. For **M4**, **M8**, and **M14**, multiple binding poses corresponding to different metabolic pathways were shown. Residues in contact with the substrate were colored based on their types: blue for basic, red for acidic, green for polar, and white for nonpolar residues. The unit for the distance is Å. This figure was rendered using VMD^27^ (http://www.ks.uiuc.edu/Research/vmd/).

To further assess the availability of the above five residues for substrate binding to CYP2J2, molecular docking was performed to search the optimal binding pose of **M4**–**M14** (Fig. 1) in CYP2J2. For simplicity, we selected those binding conformers with the minimum distance between the proposed metabolic site(s) and the heme iron, and the results were shown in Fig. 9. Except for danazol (**M6**) where no “preferred” binding pose was found, and ketoconazole (**M8**) where Phe310 was not interacting residue in one of two binding poses, the five hydrophobic residues (Ile127, Phe310, Ala311, Val380, and Ile487) were involved in positioning all the other substrates near the active site of CYP2J2. In the following, we selectively discussed the docking results for some substrates, and compared with available experimental and simulation results.

In the present docking study, danazol (**M6**) was found to occupy the position far away from the active site. The distance between the proposed metabolic site to heme iron is 12.6 Å. Interestingly, only one binding pose as shown in Fig. 9 was identified, and either repeating the docking procedure or increasing the iterations to search more potential conformers led to the same binding pose (data not shown). Experimental data showed that danazol strongly inhibited CYP2J2-mediated astemizole (**M5**), ebastine (**M7**), and terfenadine (**M9**) metabolism in a substrate-independent manner^17^. The “preferred” binding pose identified from our docking study appeared to block any other substrates to enter the active site, which may have implications for its inhibitory mechanism.

For terfenadine (**M9**), experimental results showed that two variants of CYP2J2, P351L and P115L, had similar enzyme activity for *t*-butyl methyl hydroxylation to the wild-type CYP2J2^45^. Based on the present docking result, P351 and P115 were not involved in the interactions with terfenadine, and thus could have little effect on the metabolism of terfenadine. In addition, the G312R variant was implied to destabilize the structure of CYP2J2 by altering the heme environment^45^, and cause the loss of the catalytic activity of CYP2J2^46^. The same effect was also observed in the present CYP2J2 model, as Gly312 was near the active site and the introduction of Arg would significantly change the electrostatic potential around the heme, and consequently impair the enzyme catalytic activity and structural stability.

For the terfenadone derivatives **M10** and **M12** in Fig. 1, CYP2J2 exhibited regioselectivity of hydroxylation of the Cβ atom, and Arg117 was assumed to play a critical role^12,31^. Based on the homology model of CYP2J2, a narrow channel of access to the heme was identified, which included Ile127, Phe310, Ile376, and Val380^12^. In the present CYP2J2 model, Ile376 was found to interact with **M1** and **M3**, but not with **M4**–**M14**. And Ile487 was found to be closer to the heme than Ile376 in the present CYP2J2 model (Fig. S10). Moreover, Arg117 was found to establish hydrogen bonds with the keto group of **M12** in the previous study^12^. In the current docking study, Arg117 was in contact with **M12**, but no hydrogen bond was formed between **M12** and the contacting residues of CYP2J2. In the present CYP2J2 model, Arg117 was found to stably interact with Glu222 by forming a salt-bridge, with an average distance between the oxygen atom of the carboxylic acid group of Glu222 and the nitrogen atom of the guanidine moiety of Arg117 being 3.7 ± 0.1 Å. However, the distance from the Cβ to the heme iron (3.4 Å) was larger than the distance from the Cγ atom to the heme iron (2.8 Å), which seemed to contradict the hydroxylation preference of Cβ in **M12** by CYP2J2 according to the distance criterion. It should be noted that Cγ could still move freely around Cβ, which may blur their distance difference relative to the heme iron. Thus, we proposed that, apart from the distance measurement, the chemical property of **M12** should also be considered. Because of the delocalization effect of benzene moiety, the C-H bond strength increases from Cα to Cγ. The calculated bond order for different Cα/β/γ-H bonds in terms of the natural bond order analysis with Gaussian 16^47,48^ showed that the average bond order was 0.9781, 0.9833, and 0.9918 for Cα-H, Cβ-H, and Cγ-H bond, respectively. Therefore, although Cγ was closer to the heme iron, the Cγ-H bond was stronger than the Cβ-H bond. Taken together, it appeared that Cβ achieved a desirable compromisation between the distance and bond strength, which could contribute to its regioselectivity of the hydroxylation by CYP2J2.

The last substrate discussed is the ARA (**M14**) whose primary epoxidation site seemed difficult to predict with the homology modeled structure of CYP2J2^21^. The present docking study demonstrated that the distance between the preferred metabolic site (14, 15-) and the heme iron was shorter than other metabolic sites, which was in line with the experiment finding that the 14,15-olefin (37% of total EET products) was the preferential product metabolized by CYP2J2^6^. However, it remained difficult to directly relate the preference of other products including 8,9-EET (24%), 5,6-EET (21%), and 11,12-EET (18%)^6^ with the corresponding distances of (5.4 Å, 6.6 Å), (5.0 Å, 5.5 Å) , and (5.0 Å, 5.6 Å). Other factors such as the interacting residues and the conformations of **M14** in the active site might also contribute to the difference in the ARA regioselectivity mediated by CYP2J2, which needs further investigation.

## Discussion

In this work, a new structural model of CYP2J2 was developed based on the multiple-template homology modeling approach. Note that the sequence identity between the templates and CYP2J2 was only about 40% (Fig. S11), a long-time relaxation under more realistic conditions seemed necessary. Represented with the improved CHARMM36m force field parameters^49^, the CYP2J2 model was fully relaxed in explicit solvent by running 500-ns MD simulations. To assess the sensitivity of this model to substrates, three chemically similar probes **M1**–**M3** were docked to the representative conformation of CYP2J2, and the obtained initial binding poses were subjected to 500-ns MD simulations. The simulation results suggested that **M1**–**M3** tended to adopt extended conformations in the binding pocket of CYPL2J2, similar to their behavior in solvent. The induced-fit binding of these probes enabled **M2** and **M3** to change their conformations until a favorable binding pose was adopted by each of them. The highest binding affinity predicted for **M2** may have implications for its strongest fluorescence response to CYP2J2. Besides the distance measurement to dictate the catalytic reactivity, the present results highlighted the important role of electrostatic potential around the substrates in predicting the catalytic efficiency of CYP2J2. We also showed that structurally similar **M1**–**M3** resulted in divergent conformational dynamics of CYP2J2, and such conformational diversity may contribute to its capability of accommodating and metabolizing substrates with varying sizes and shapes.

From the interactions of **M1**–**M3** with CYP2J2, five hydrophobic residues (Ile127, Phe310, Ala311, Val380, and Ile487), were identified to restrict **M1**–**M3** access to the heme. Compared to the conformation of free CYP2J2, the hydrophobic clamp formed by these residues seemed more stable in the interactions of **M2** with CYP2J2, suggesting that maintenance of this topology maybe related to the catalytic property of CYP2J2. To confirm that these residues acted as molecular determinant for substrate binding and specificity, a benchmark testing was carried out for a set of 11 substrates of CYP2J2 (**M4**–**M14**). Except for danazol (**M6**) and one of binding poses of ketoconazole (**M8**) where Phe310 was not involved, the five key residues were all in contact with the bound substrates. Although the present docking studies predicted the preferred binding pose(s) for these substrates, it remained challenging to predict the regioselectivity of substrates such as terfenadone derivative (**M12**) and ARA (**M14**) based on docking with single conformation, as well the distance criterion used to evaluate the catalytic reactivity. The physico-chemical property of a substrate obtained by quantum calculations could also be useful to elucidate the substrate metabolism.

The present CYP2J2 model provided a structural basis for the investigation of the metabolism of other endogenous or exogenous compounds. It should be noted that most reported CYPs crystal structures and structural models of CYP2J2 lack the N-terminal transmembrane helix, whereas the truncated forms preserve the catalytic activity of the enzymes^50^. In addition to function in a soluble form, CYPs are able to attach to membrane with its N-terminal, and interact with its redox partner CYP reductase in the membrane environment, which are essential for electron transfer in the catalytic cycle^51^. Interactions with membrane have been observed to modulate the conformational dynamics of human aromatase (CYP19A1) and alter the substrate/inhibitor access channels^52^. Computational simulations and experimental data have also revealed that interactions of CYP enzymes with partner reductase in the membrane could affect the substrate binding^53,54^. With the addition of the N-terminus of CYP2J2, the present structural model of CYP2J2 could be applied to study its interactions with membranes, as well as interactions with its partner in membranes.

## Methods

### Homology modeling and model relaxation

The homology model of CYP2J2 was constructed based on the following templates: CYP2D6 (PDB ID: 2F9Q)^55^, CYP2A6 (PDB ID: 1Z10)^56^, CYP2B4 (PDB ID: 2BDM)^57^, CYP2C9 (PDB ID: 1OG5)^58^, and CYP2C8 (PDB ID: 1PQ2)^59^. These CYP2 enzymes display similar sequence identity (40.9%–42.6%) with that of CYP2J2 (Fig. S11). The multiple sequence alignment was carried out using the Clustal Omega web server^60^, and the result was shown in Fig. S12. The homology modeling was performed using the Modeller software (v9.17)^61,62^, and the model built with the lowest DOPE score was selected for further relaxation.

The model structure of CYP2J2 was fully relaxed by running 500-ns MD simulations in aqueous solution. The intramolecular interactions were represented with the CHARMM36m force field parameters^49^. The protein was solvated by the TIP3P water model in a rectangular box with the minimum distance between the protein and the box boundary being 15 Å. The salt concentration (NaCl) of the system was 0.15 M. The particle mesh Ewald method was used to treat long-range electrostatic interactions^63^. The system was minimized for 5000 steps, and equilibrated for 50 ps with the backbone and sidechain atoms restrained with a force constant of 400 kJ/(mol·nm^2^) and 40 kJ/(mol·nm^2^), respectively. The system temperature was kept at 303 K using the Nosé–Hoover thermostat^64^. The 500-ns production simulation was performed in the NPT ensemble at 303 K and 1 bar without restraints. The time-step was 2 fs and the pressure was maintained at 1 bar using the Parrinello-Rahman method^65^. MD simulations were performed using the GROMACS program (version 2018.4)^66,67^. The CHARMM-GUI web server was used to generate the input files for all simulations^68,69^.

### Molecular docking and MD simulation of protein–ligand interactions

The representative conformation of CYP2J2 was selected from the last 100-ns trajectory. The average structure of CYP2J2 was first calculated from the last-100 ns trajectory. As this average conformation may not represent the real conformation of CYP2J2, we used this average structure as a reference to calculate the RMSDs for the conformations from the last 100-ns trajectory, and selected the conformation with the minimum RMSD. If two conformations showed the same RMSD, the conformation with the lower energy (see conformational energy calculation below) was selected and used for molecular docking. The representative conformation of CYP2J2 was shown in Fig. 2. The PROCHECK program^70^ was used to assess the stereochemical quality of the protein structure. Nearly all residues (99.5%) of the CYP2J2 model were found in the allowed regions in Ramchandran plot, and 369 residues (91.1%) whose Φ-Ψ angles were in the most favored regions (Fig. S14), suggesting a good quality model.

The molecular structure of **M1**/**M2**/**M3** was represented with the CHARMM General Force Field (CGenFF)^71^ along with the calculated RESP charges (Fig. S13)^72^. The structure of CYP2J2 was represented with the CHARMM36m force field parameters^49^. Molecular docking was performed to search the favorable binding poses of **M1**/**M2**/**M3** in the structure of CYP2J2. Autodock 4.2 program^73,74^ was applied to dock **M1**/**M2**/**M3** to the ligand binding pocket above the heme motif. In the docking process, the CYP2J2 structure was modeled as rigid while the ligand structure was modeled as flexible. Lamarckian genetic algorithm^75^ was applied to search a total of 100 potential binding poses in CYP2J2. The optimal ligand conformer was selected based on the following criteria: (1) The distance between the metabolic site of the ligand and the heme iron atom should be as short as possible; and (2) and if there were multiple conformers showing the same minimum distance, the conformer with the lower binding energy estimated in terms of the docking score was selected. Molecular docking of **M4**–**M14** to the binding site of CYP2J2 was also performed.

The initial binding conformers of **M1**–**M3** were shown in Figs. 3–5, respectively. For **M2**, a second binding conformer that was similar to **M1** was also chosen. For **M3**, the present docking study was unable to find the preferred binding pose that displayed the minimum distance between the metabolic site and the heme iron, which was probably because the binding pocket in the representative conformation of CYP2J2 was not large enough to accommodate **M3** in the extended conformation. Therefore, we selected a binding conformer of **M3** that was similar to the second binding pose of **M2** (Fig. S2). MD simulations of each protein–ligand complex were performed following the same procedure as used in the simulations of CYP2J2. The production simulations lasted for 500 ns for each system, and the last 100- or 50-ns trajectory was used for further analyses. Note that in both docking and MD simulations, we used the same CHARMM force field parameters^49^ for protein and CGenFF parameters^71^ (with the RESP partial charges) for the ligands.

### Evaluation of protein stability and protein–ligand binding affinity

The relative stability of CYP2J2 in complex with **M1**/**M2**/**M3** was evaluated by the conformational energy, which was calculated using the generalized Born using molecular volume (GBMV) implicit solvent model implemented in the CHARMM (v44b1) program^76^. The single point energy was calculated after a 200-step minimization of each conformation using the GBMV II algorithm^77,78^. Other energy terms including bonded energy, van der Waals energy, electrostatic energy, and solvation energy were also obtained with the GB implicit solvent model. The block average method was used to estimate the mean values and standard deviations.

The binding free energy of **M1**/**M2**/**M3** to CYP2J2 was calculated using the *g_mmpbsa* program^79^ which implements the molecular mechanics Poisson–Boltzmann surface area (MM-PBSA) method^80,81^ to predict the binding affinity for protein–ligand complex. The entropy contribution was not included in the current binding energy calculations. The default bootstrap method was used to estimate the mean values and standard deviations.

## Supplementary Information

Supplementary Information 1.

Supplementary Information 2.

Supplementary Information 3.

## Data Availability

The datasets generated and analyzed during the current study are available from the corresponding author on reasonable request. Structures of the modeled CYP2J2 (in pdb format) and M1–M14 (in mol2 format) are available with the published manuscript.
