# Supplementary material for: Molecular determinant of substrate binding and specificity of cytochrome P450 2J2
Source: Sci Rep. 2020 Dec 17;10:22267. doi: 10.1038/s41598-020-79284-0 (PMC7746748; doi:10.1038/s41598-020-79284-0)
Supplement: Supplementary file 3 — Supplementary Information 3. [file 41598_2020_79284_MOESM3_ESM.pdf]

Supplementary Information

Molecular Determinant of Substrate Binding and Specificity of Cytochrome P450 2J2

Liang Xu* and Liao Y. Chen

Department of Physics and Astronomy, University of Texas at San Antonio, One UTSA Circle, San Antonio, TX, 78249, USA

E-mail: liang.xu@utsa.edu.

Contents

Fig. S1. RMSD and RMSF for the free and ligand-bound CYP2J2……………………………..Page S2
Fig. S2. Overlapping of the initially folded conformation of **M2** and **M3** in CYP2J2…………..Page S3
Fig. S3. Conformational changes of **M3** in CYP2J2……………………………………………..Page S4
Fig. S4. A conformation of **M2** in the active site of CYP2J2 with a bound water molecule…….Page S4
Fig. S5. Overlapping of conformations of **M1**–**M3** in solvent and in CYP2J2…………………..Page S5
Fig. S6. Electrostatic potential surface calculated for the starting conformation of CYP2J2……Page S6
Fig. S7. Electrostatic potential surface for a randomly selected conformation of CYP2J2……..Page S7
Fig. S8. Substrate access and exit channels in different systems……………………………..Page S8
Fig. S9. The topology of the channel formed by residues I127, F310, A311, V380, and I487.....Page S9
Fig. S10. The positons of I375, I376, and I487 relative to the heme plane…………………….Page S9
Fig. S11. Sequence identity between CYP2J2 and the templates………………………………Page S10
Fig. S12. Multiple sequence alignment of CYP2J2 and the correspoding templates……………Page S11
Fig. S13. RESP charges calculated for **M1**–**M3**……………………………………………..Page S12
Fig. S14. Ramachandran plot of the CYP2J2 model……………………………………………Page S13
References……………………………………………………………………………………Page S14

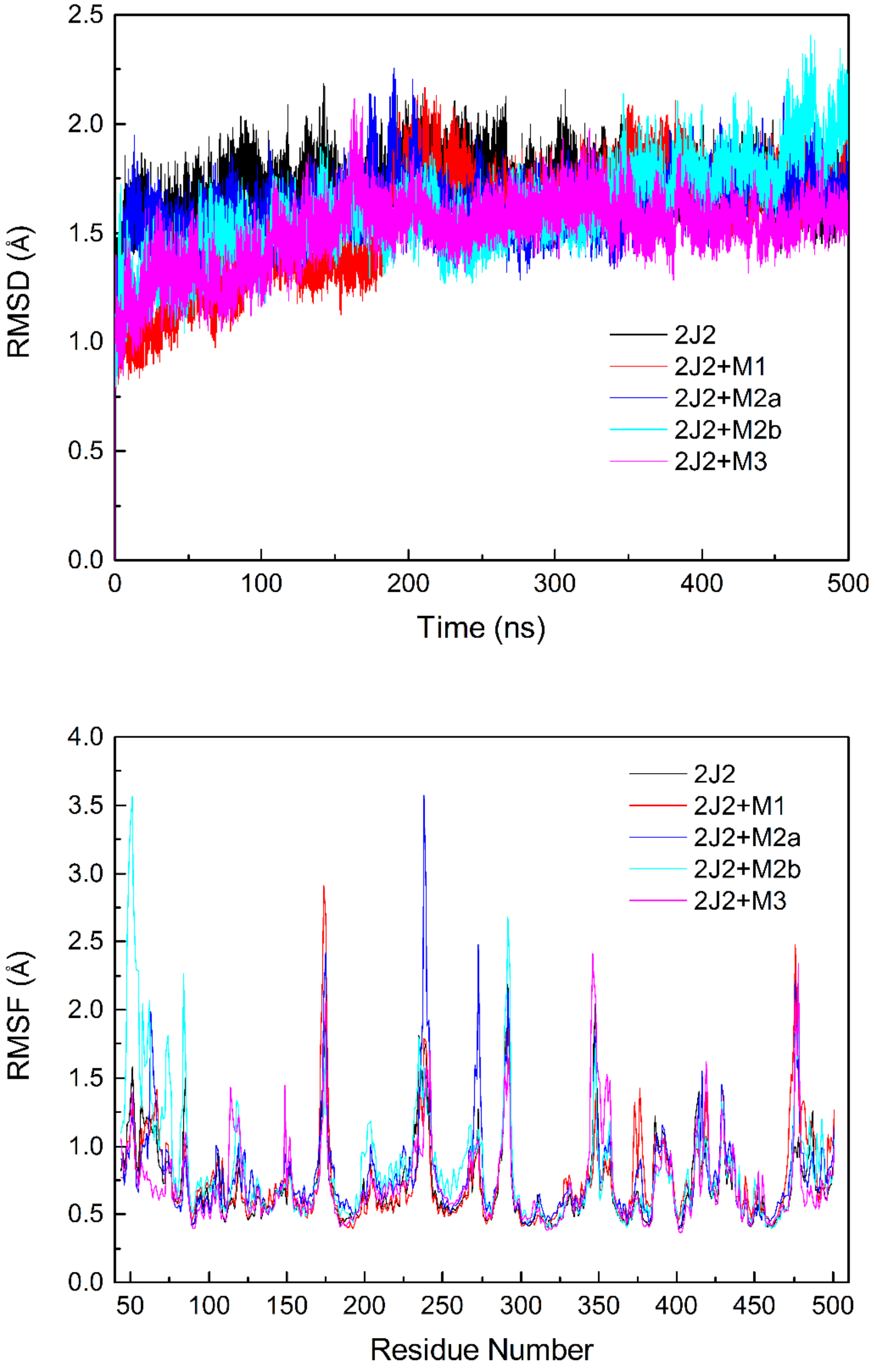

Fig. S1. RMSD and RMSF for the free and ligand-bound CYP2J2. **M2**a and **M2**b denote the initially extended (M2a) and folded (M2b) binding poses of **M2** in the binding pocket of CYP2J2.

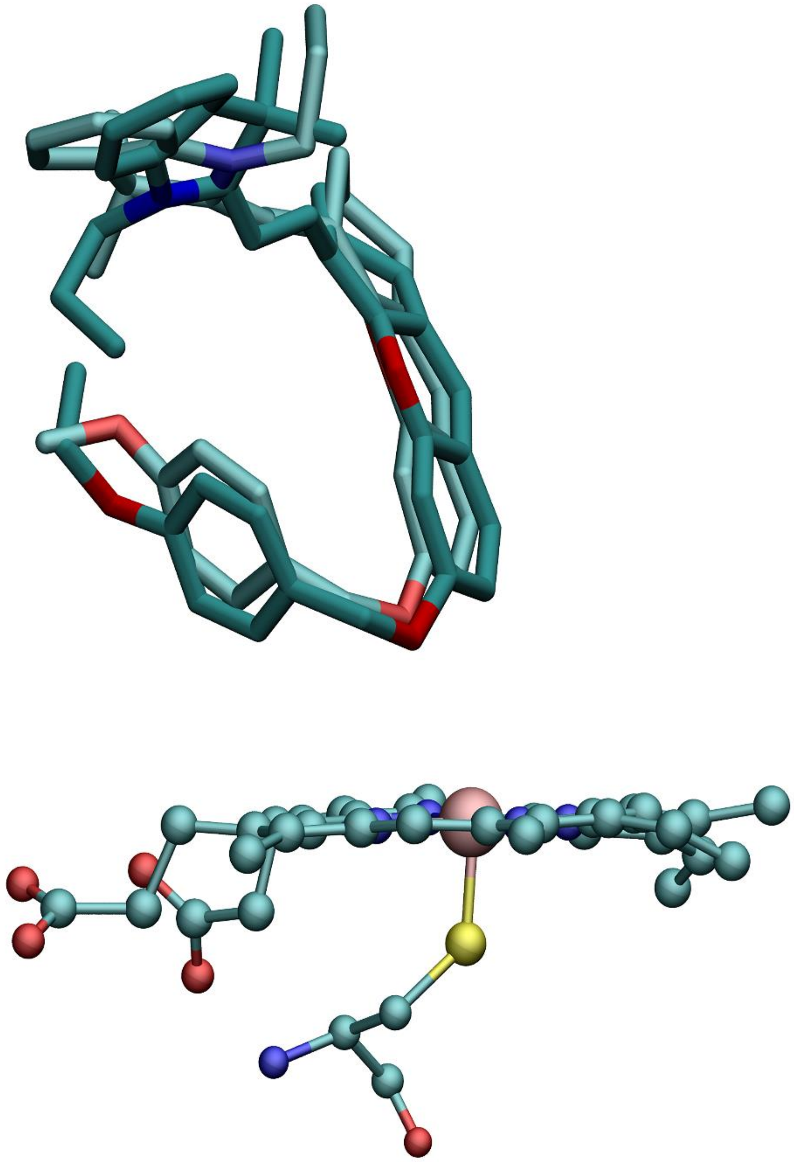

Fig. S2. Overlapping of the initially folded conformation of **M2** and **M3** in CYP2J2. This figure was rendered using VMD[1] (http://www.ks.uiuc.edu/Research/vmd/).

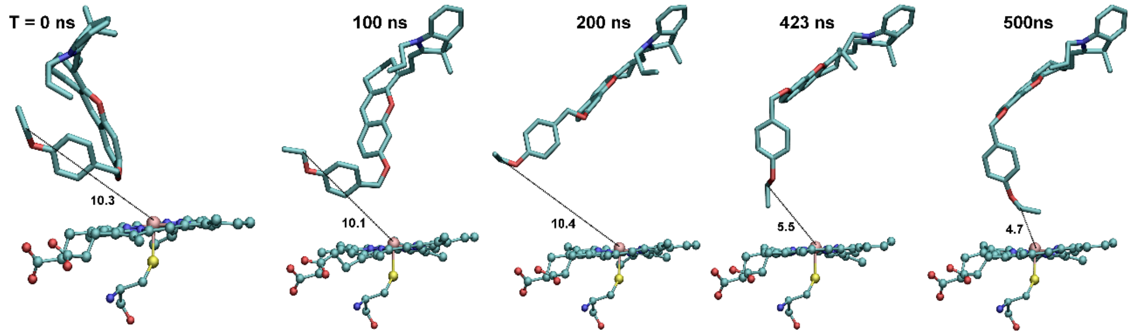

Fig. S3. Conformational changes of **M3** in the binding pocket of CYP2J2 during MD simulations. The distance unit is Å. This figure was rendered using VMD[1] (http://www.ks.uiuc.edu/Research/vmd/).

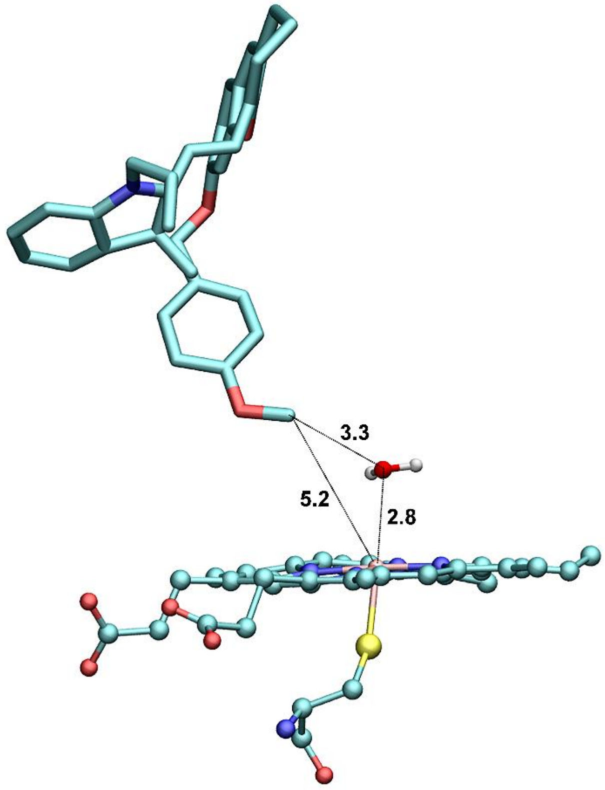

Fig. S4. A representative conformation of **M2** in the active site of CYP2J2 with a transiently bound water molecule between **M2** and the heme moiety. The distance unit is Å. This figure was rendered using VMD[1] (http://www.ks.uiuc.edu/Research/vmd/).

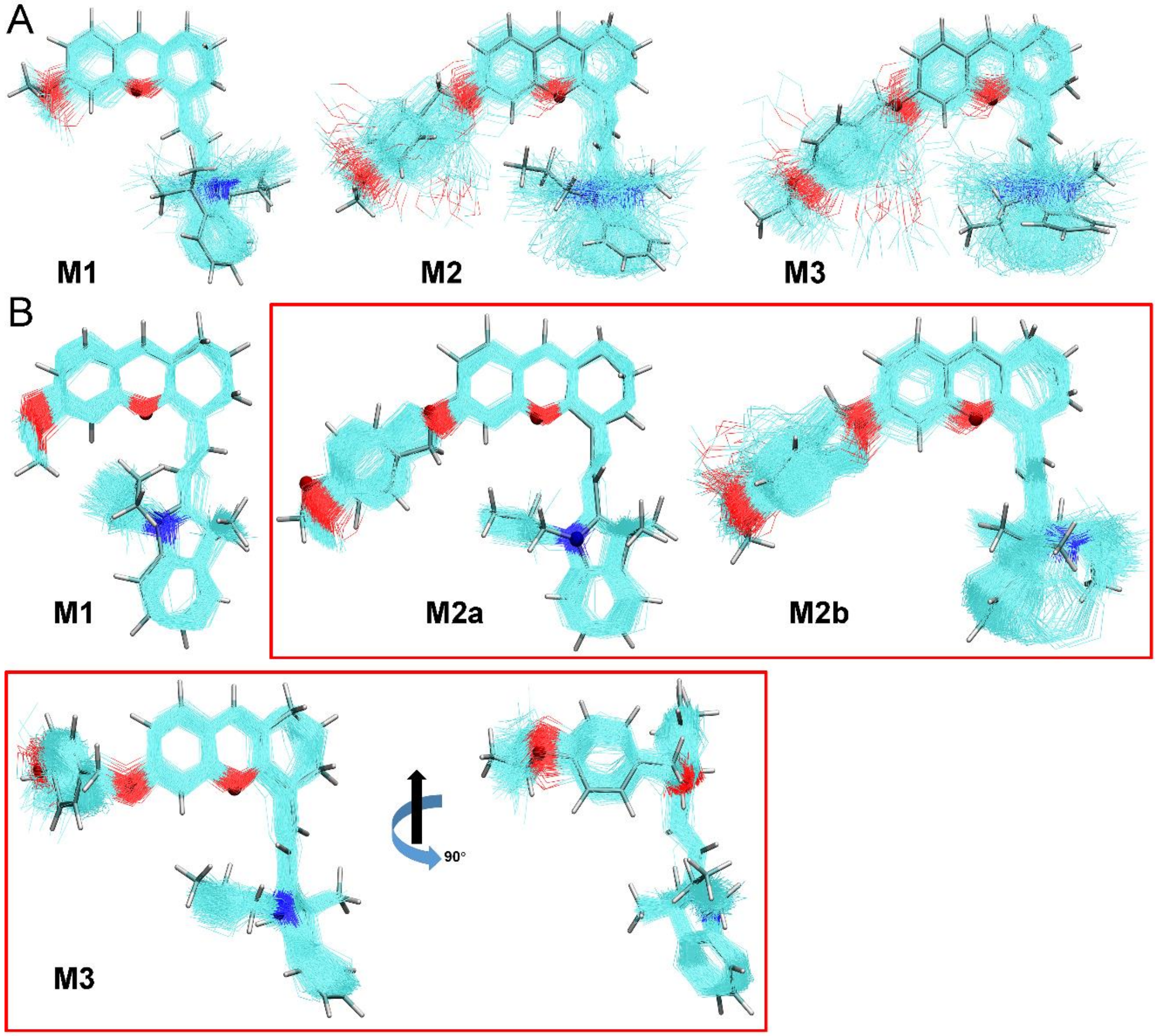

Fig. S5. Overlapping of conformations of **M1**–**M3** sampled in solvent (A) and in the active site of CYP2J2 (B). **M2**a and **M2**b denote the initially extended (M2a) and folded (M2b) binding poses of **M2** in the binding pocket of CYP2J2. Large strain energy was induced due to the deformed conformation of **M3** in the binding site of CYP2J2. This figure was rendered using VMD[1] (http://www.ks.uiuc.edu/Research/vmd/).

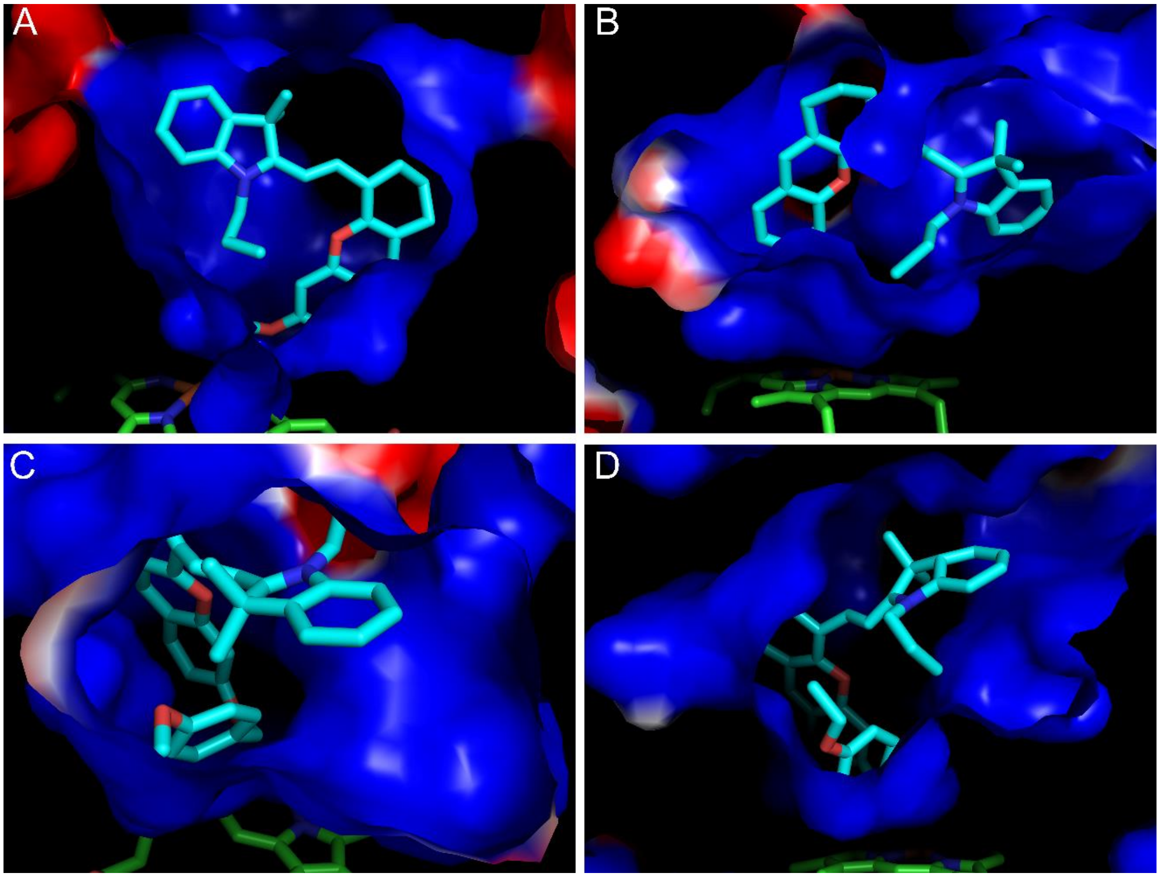

Fig. S6. Electrostatic potential surface calculated for the starting conformation of CYP2J2 in complex with **M1** (A), **M2** in the initially extended (B) and folded (C) conformation, and **M3** (D). Blue color indicates positive electrostatic potentials, and red color indicates negative electrostatic potentials. This figure was rendered using PyMOL[2].

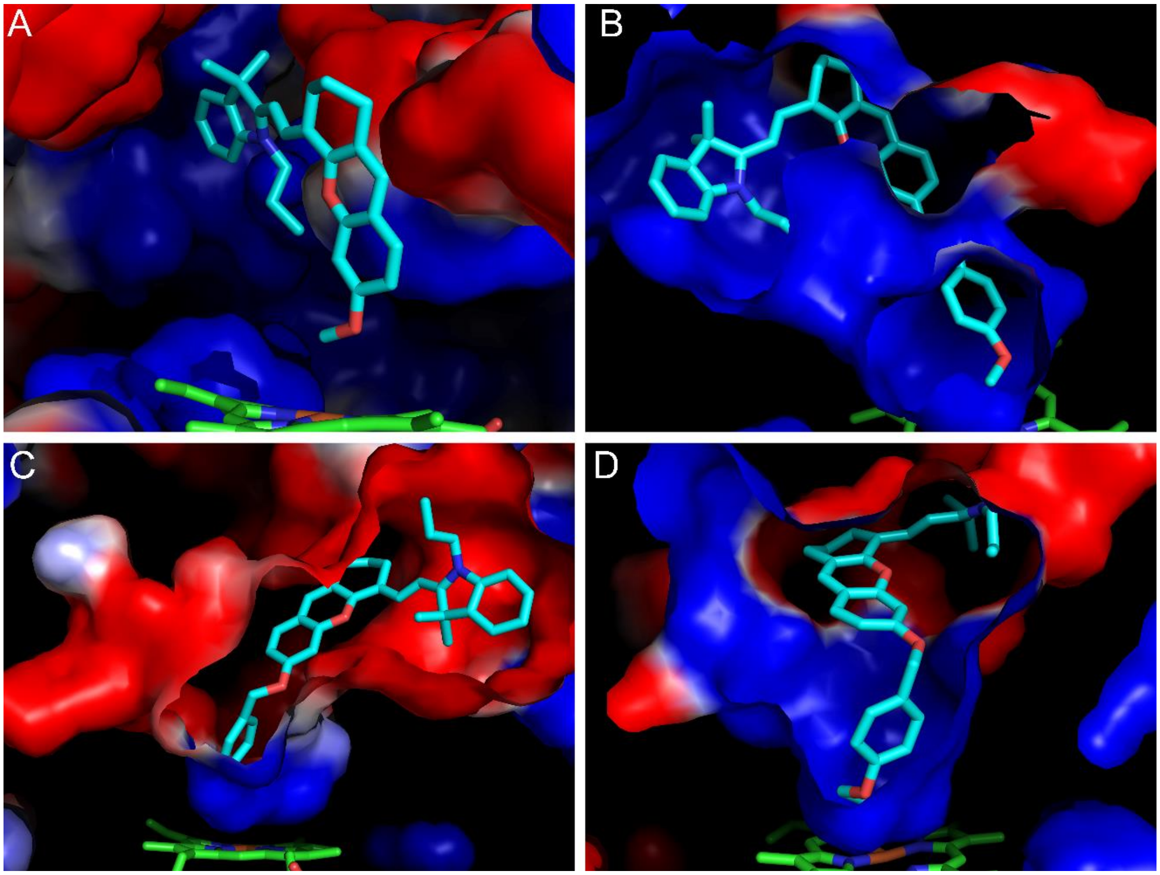

Fig. S7. Electrostatic potential surface calculated for a randomly selected conformation of CYP2J2 in complex with **M1** (A), **M2** in the initially extended (B) and folded (C) conformation, and **M3** (D). Blue color indicates positive electrostatic potentials, and red color indicates negative electrostatic potentials. This figure was rendered using PyMOL[2].

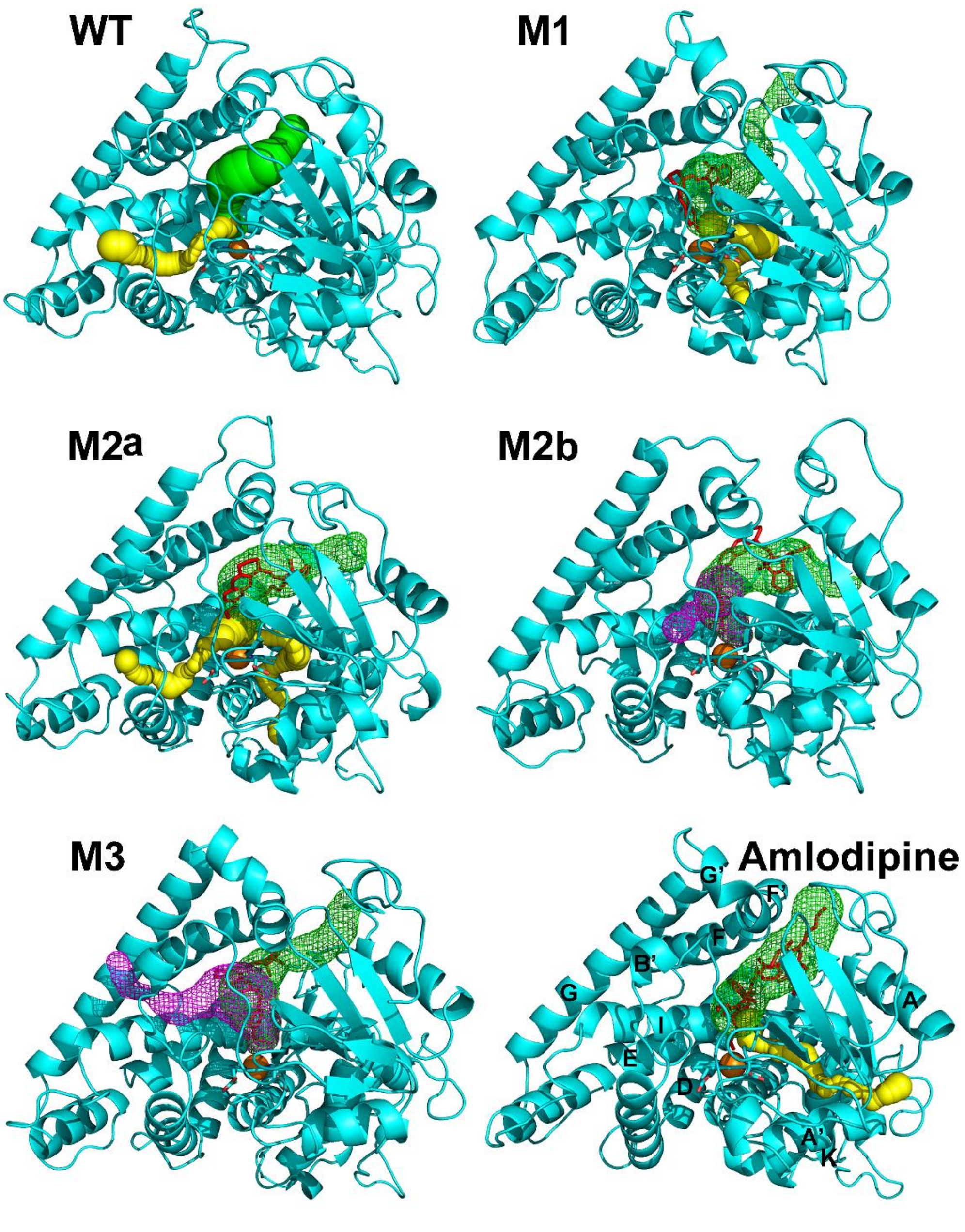

Fig. S8. Substrate access and exit channels in different systems of CYP2J2 in complex with M1–M3. **WT** denotes the ligand-free CYP2J2, corresponding to the representative conformation showin in Fig. 2. M2a and M2b denote initially extended and folded conformation of **M2**, respectively. These four conformations (M1, M2a, M2b, and M3) are the same conformations of CYP2J2 shown in Fig. 7. For comparison, the substrate access/exit channels in the human CYP2B6 in complex with two amlodipine molecules (PDB ID: 3UA5) were also calculated. The optimal exit channel was assumed to overlap with the access channel as little as possible, and shown in yellow spheres. If no such exit channel was detected, the channel overlapping the least with the access channel was shown in magenta mesh representation. This figure was rendered using PyMOL[2].

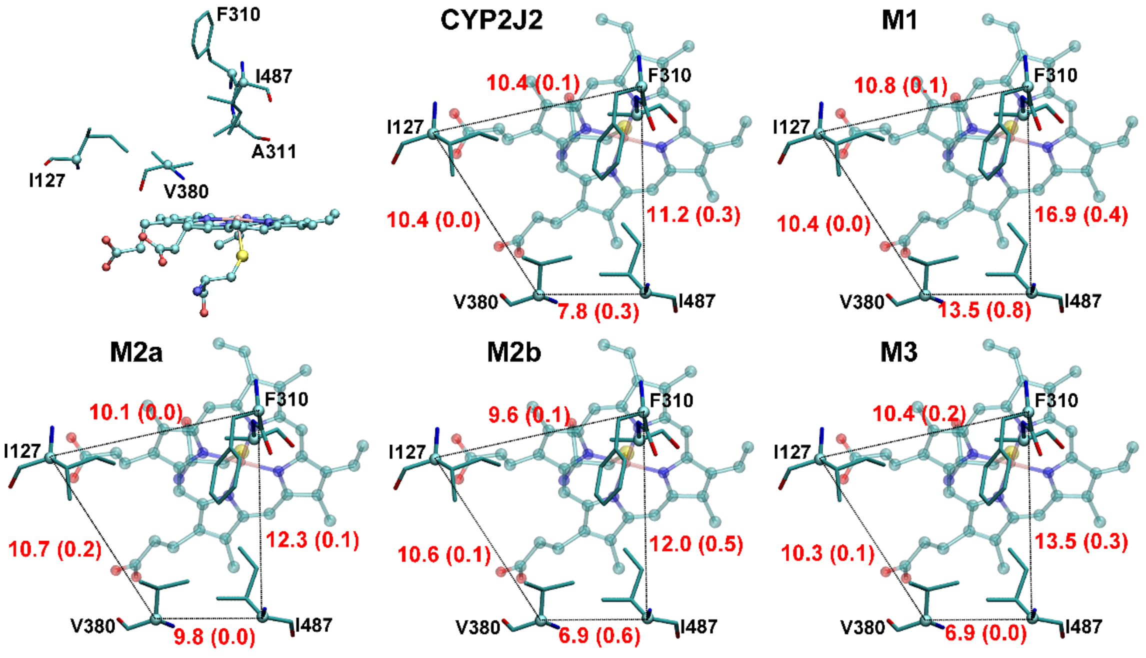

Fig. S9. The topology of the channel formed by key residues Ile127, Phe310, Ala311, Val380, and Ile487. The distance between two residues was block-averaged over the last 100-ns trajectory of each system. The unit for the distance is Å. For simplicity, the same conformation was used to label the distances in different systems. **M2**a and **M2**b denote the initially extended (M2a) and folded (M2b) binding poses of **M2** in the binding pocket of CYP2J2. This figure was rendered using VMD[1] (http://www.ks.uiuc.edu/Research/vmd/).

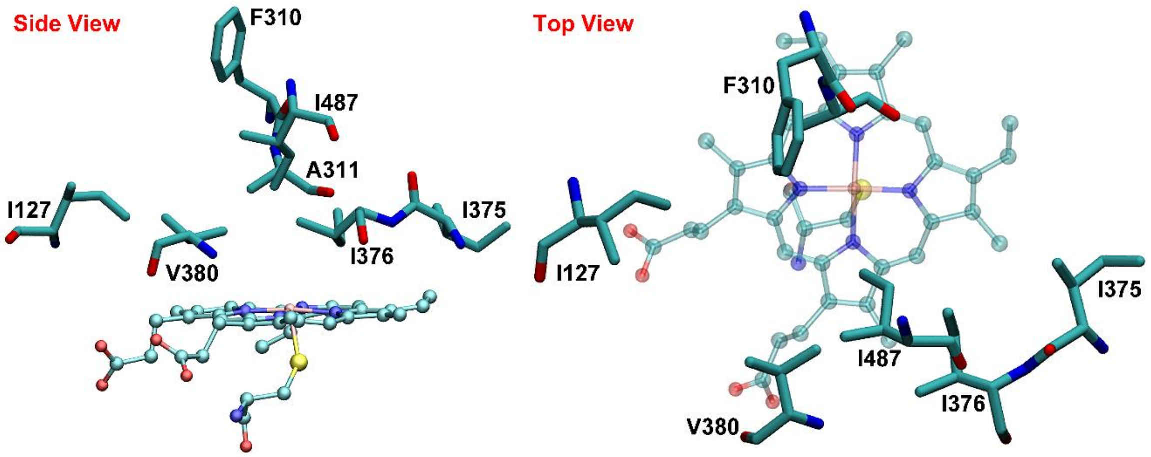

Fig. S10. The positons of Ile375, Ile376, and Ile487 relative to the heme plane. Residue Ile487 is closer to the heme than Ile375 and Ile376. This figure was rendered using VMD[1] (http://www.ks.uiuc.edu/Research/vmd/).

	2F9Q	2J2X	2BDM	1Z10	1OG5	1PQ2
1: 2F9Q	100.00	42.60	42.09	37.64	41.20	42.09
2: 2J2X	42.60	100.00	42.42	40.91	41.27	41.52
3: 2BDM	42.09	42.42	100.00	53.88	51.19	54.00
4: 1Z10	37.64	40.91	53.88	100.00	49.67	49.89
5: 1OG5	41.20	41.27	51.19	49.67	100.00	78.74
6: 1PQ2	42.09	41.52	54.00	49.89	78.74	100.00

Fig. S11. Sequence identity between CYP2J2 and the templates used to model the structure of CYP2J2.

```
2J2X: MLAAMGSLAAALWAVVHPRTLLLGTVAFLLAADFLKRRRPKNYPPGPWRLPFLGNFFLVD 60
2F9Q: --------------------------------MAKKTSSKGKLPPGPLPLPGLGNLLHVD 28
1Z10: --------------------------------MAKKTSSKGKLPPGPTPLPFIGNYLQLN 28
2BDM: --------------------------------MAKKTSSKGKLPPGPSPLPVLGNLLQMD 28
1OG5: --------------------------------MAKKTSSKG-RPPGPTPLPVIGNILQIG 27
1PQ2: --------------------------------MAKKTSSKGKLPPGPTPLPIIGNMLQID 28
                                         *       ****  ** :** : :.

2J2X: FEQSHLEVQLFVKKYGNLFSLELGDISAVLITGLPLIKEALIHMDQNFGNRPVTPMREHI 12C
2F9Q: FQNTPYCFDQLRRRFGDVFSLQLAWTPVVVLNGLAAVREALVTHGEDTADRPPVPITQIL 88
1Z10: TEQMYNSLMKISERYGPVFTIHLGPRRVVVLCGHDAVREALVDQAEEFSGRGEQATFDWV 88
2BDM: RKGLLRSFLRLREKYGDVFTVYLGSRPVVVLCGTDAIREALVDQAEAFSGRGKIAVVDPI 88
1OG5: IKDISKSLTNLSKVYGPVFTLYFGLKPIVVLHGYEAVKEALIDLGEEFSGRGIFPLAERA 87
1PQ2: VKDICKSFTNFSKVYGPVFTVYFGMNPIVVFHGYEAVKEALIDNGEEFSGRGNSPISQRI 88
       :     .  : . :* :*:: :.    *:: *   ::***:   :  ..*      :

2J2X: FKKNG----LIMSSGQAWKEQRRFTLTALRNFGLGKKSLEERIQEEAQHLTEAIKEENGQ 176
2F9Q: GFGPRSQGVFLARYGPAWREQRRFSVSTLRNLGLGKKSLEQWVTEEAACLCAAFANHSGR 148
1Z10: FKGYG----VVFSNGERAKQLRRFSIATLRDFGVGKRGIEERIQEEAGFLIDALRGTGGA 144
2BDM: FQGYG----VIFANGERWRALRRFSLATMRDFGMGKRSVEERIQEEARCLVEELRKSKGA 144
1OG5: NRGFG----IVFSNGKKWKEIRRFSLMTLRNFGMGKRSIEDRVQEEARCLVEELRKTKAS 143
1PQ2: TKGLG----IISSNGKRWKEIRRFSLTTLRNFGMGKRSIEDRVQEEAHCLVEELRKTKAS 144
               .:   *   :  ***:: ::*::*:**:.:*: : ***  *   :    .

2J2X: PFDPHFKINNAVSNIICSITFGERFEYQDSWFQQLLKLLDEVTYLEASKTCQLYNVFPWI 236
2F9Q: PFRPNGLLDKAVSNVIASLTCGRRFEYDDPRFLRLLDLAQEGLKEESGFLREVLNAVP-V 207
1Z10: NIDPTFFLSRTVSNVISSIVFGDRFDYKDKEFLSLLRMMLGIFQFTSTSTGQLYEMFSSV 204
2BDM: LLDNTLLFHSITSNIICSIVFGKRFDYKDPVFLRLLDLFFQSFSLISSFSSQVFELFSGF 204
1OG5: PCDPTFILGCAPCNVICSIIFHKRFDYKDQQFLNLMEKLNENIEILSSPWIQVYNNFPAL 203
1PQ2: PCDPTFILGCAPCNVICSVVFQKRFDYKDQNFLTLMKRFNENFRILNSPWIQVCNNFPLL 204
             :    .*:*.*:    **:*.*  *  *:               :: : .  .

2J2X: MKFLPGPHQTLFSNWKKLKLFVSHMIDKHRKDWNPA-ETRDFIDAYLKEMSKHTGNPTSS 295
2F9Q: DRHIPALAGKVLRFQKAFLTQLDELLTEHRMTWDPAQPPRDLTEAFLAEMEKAKGNPESS 267
1Z10: MKHLPGPQQQAFQLLQGLEDFIAKKVEHNQRTLDPN-SPRDFIDSFLIRMQEEEKNPNTE 263
2BDM: LKYFPGTHRQIYRNLQEINTFIGQSVEKHRATLDPS-NPRDFIDVYLLRMEKDKSDPSSE 263
1OG5: LDYFPGTHNKLLKNVAFMKSYILEKVKEHQESMDMN-NPQDFIDCFLMKMEKEKHNQPSE 262
1PQ2: IDCFPGTHNKVLKNVALTRSYIREKVKEHQASLDVN-NPRDFIDCFLIKMEQEKDNQKSE 263
         :*.               : . : .::   :     :*: : :* .*.:   : :.

2J2X: FHEENLICSTLDLFFAGTETTSTTLRWALLYMALYPEIQEKVQAEIDRVIGQGQQPSTAA 355
2F9Q: FNDENLRIVVADLFSAGMVTTSTTLAWGLLLMILHPDVQRRVQQEIDDVIGQVRRPEMGD 327
1Z10: FYLKNLVMTTLNLFIGGTETVSTTLRYGFLLLMKHPEVEAKVHEEIDRVIGKNRQPKFED 323
2BDM: FHHQNLILTVLSLFFAGTETTSTTLRYGFLLMLKYPHVTERVQKEIEQVIGSHRPPALDD 323
1OG5: FTIESLENTAVDLFGAGTETTSTTLRYALLLLLKHPEVTAKVQEEIERVIGRNRSPCMQD 322
1PQ2: FNIENLVGTVADLFVAGTETTSTTLRYGLLLLLKHPEVTAKVQEEIDHVIGRHRSPCMQD 323
      *  :.*   . .** .*  *.**** :.:* :  :*.:  :*: **: ***  : *

2J2X: RESMPYTNAVIHEVQRMGNIIPLNVPREVTVDTTLAGYHLPKGTMILTNLTALHRDPTEW 415
2F9Q: QAHMPYTTAVIHEVQRFGDIVPLGMTHMTSRDIEVQGFRIPKGTTLITNLSSVLKDEAVW 387
1Z10: RAKMPYMEAVIHEIQRFGDVIPMSLARRVKKDTKFRDFFLPKGTEVYPMLGSVLRDPSFF 383
2BDM: RAKMPYTDAVIHEIQRLGDLIPFGVPHTVTKDTQFRGYVIPKNTEVFPVLSSALHDPRYF 383
1OG5: RSHMPYTDAVVHEVQRYIDLLPTSLPHAVTCDIKFRNYLIPKGTTILISLTSVLHDNKEF 382
1PQ2: RSHMPYTDAVVHEIQRYSDLVPTGVPHAVTTDTKFRNYLIPKGTTIMALLTSVLHDDKEF 383
      :  ***  **:**:**  :::* .: : .. *  . .: :**.* :   * :  :*   :

2J2X: ATPDTFNPDHFLE-NGQFKKREAFMPFSIGKRACLGEQLARTELFIFFTSLMQKFTFRPP 474
2F9Q: EKPFRFHPEHFLDAQGHFVKPEAFLPFSAGRRACLGEPLARMELFLFFTSLLQHFSFSVP 447
1Z10: SNPQDFNPQHFLNEKGQFKKSDAFVPFSIGKRNCFGEGLARMELFLFFTTVMQNFRLKSS 443
2BDM: ETPNTFNPGHFLDANGALKRNEGFMPFSLGKRICLGEGIARTELFLFFTTILQNFSIASP 443
1OG5: PNPEMFDPHHFLDEGGNFKKSKYFMPFSAGKRICVGEALAGMELFLFLTSILQNFNLKSL 442
1PQ2: PNPNIFDPGHFLDKNGNFKKSDYFMPFSAGKRICAGEGLARMELFLFLTTILQNFNLKSV 443
       .*  *.* ***:  * : : . *:*** *:* * ** :*  ***:*:*:::*:* :

2J2X: NNEKLS--LKFRMGITISPVSHRLCAVPQV---  502
2F9Q: TGQPRPSH-HGVFAFLVSPSPYELCAVPRHHHH  479
1Z10: QSPKDIDVSPKHVGFATIPRNYTMSFLPRHHHH  476
2BDM: VPPEDIDLTPRESGVGNVPPSYQIRFLARHHHH  476
1OG5: VDPKNLDTTPVVNGFASVPPFYQLCFIPVHHHH  475
1PQ2: DDLKNLNTTAVTKGIVSLPPSYQICFIPVHHHH  476
                   ..   *  : :  :
```

Fig. S12. Multiple sequence alignment of CYP2J2 and the correspoding templates.

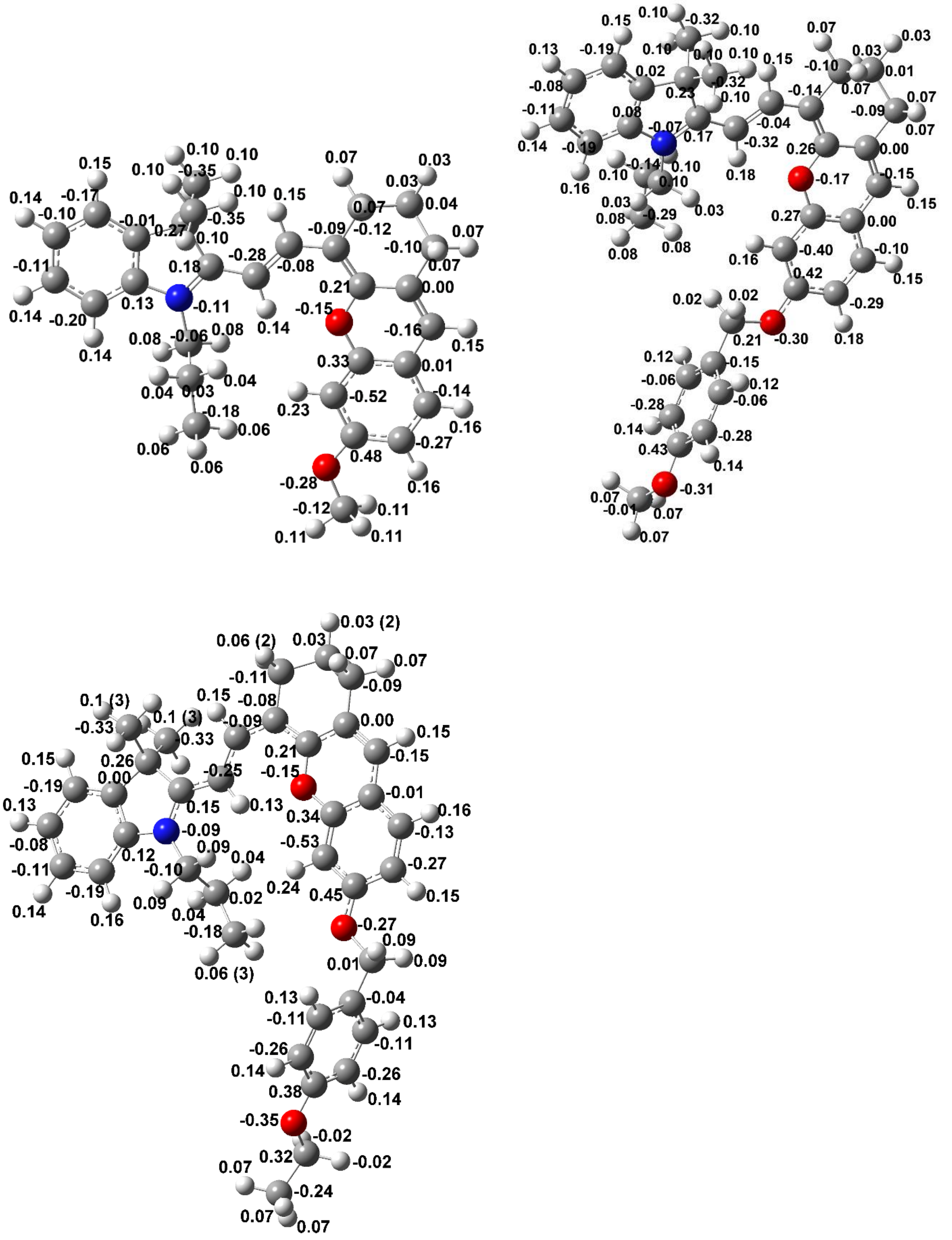

Fig. S13. RESP charges calculated for **M1**–**M3**. This figure was rendered using GaussView 5.0[4].

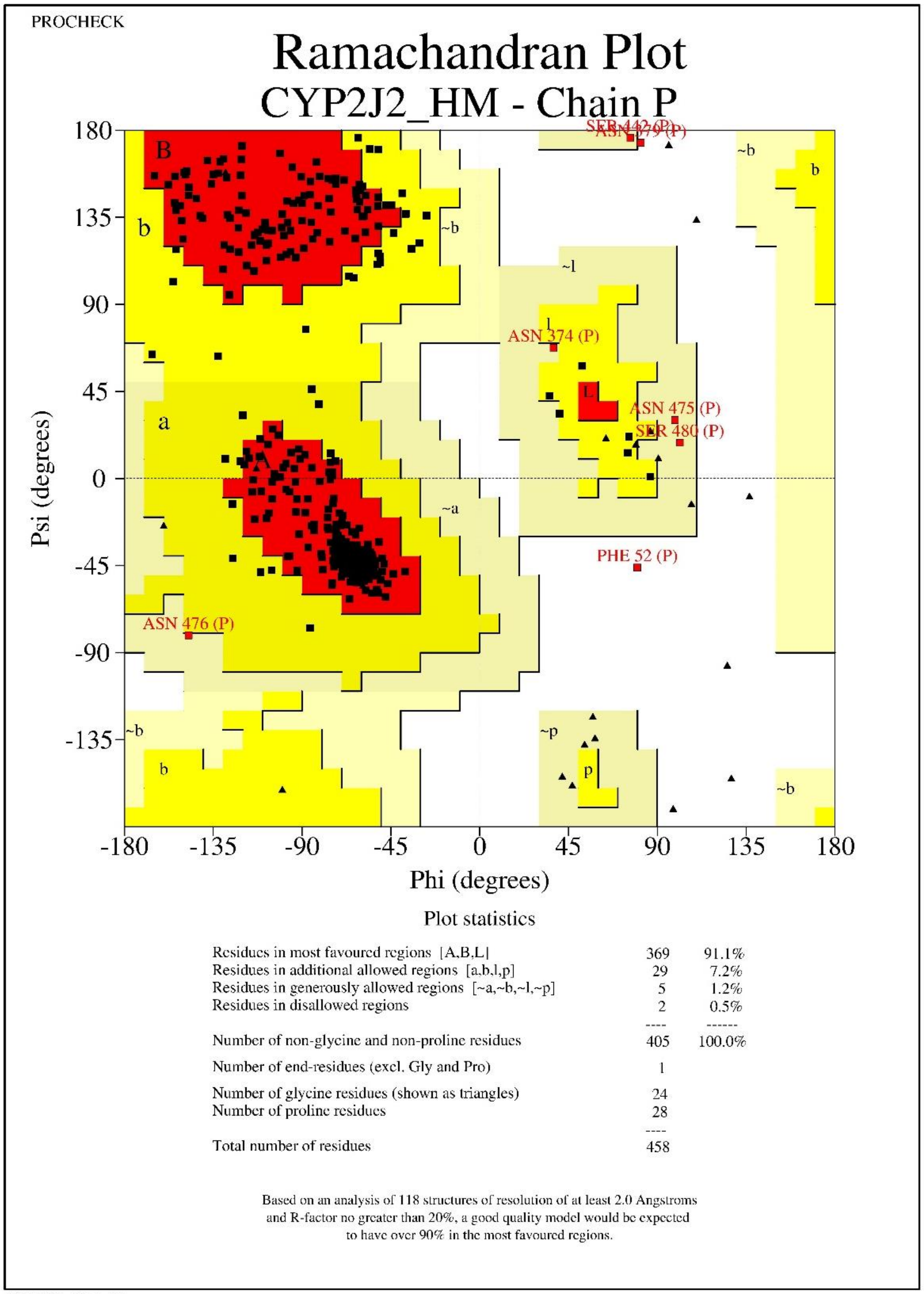

CYP2J2_HM_01.ps

Fig. S14. The Ramachandran plot of the CYP2J2 model. This figure was rendered using PROCHECK[3].

References

1. Humphrey, W., Dalke, A. & Schulten, K. VMD: Visual molecular dynamics. *Journal of Molecular Graphics* **14**, 33-38, doi:10.1016/0263-7855(96)00018-5 (1996).
2. The PyMOL Molecular Graphics System, Version 1.8.x, Schrödinger, LLC.
3. Laskowski, R. A., MacArthur, M. W., Moss, D. S. & Thornton, J. M. PROCHECK: a program to check the stereochemical quality of protein structures. *Journal of Applied Crystallography* **26**, 283-291, doi:10.1107/s0021889892009944 (1993).
4. Nielsen, A.B. & Holder, A.J. Gauss View 5.0, User's Reference. GAUSSIAN Inc., Pittsburgh, (2009).
